# Hippo signaling pathway in cervical cancer: insights into mechanisms and therapeutic potential

**DOI:** 10.3389/fonc.2025.1662499

**Published:** 2025-11-03

**Authors:** Yanmei Sun, Fei Zhou, Xiuhong Zhong, Xiatong Lv, Yue Liu, Yi Zhang, Ryan D. Fine, Mingguang Li

**Affiliations:** ^1^ School of Laboratory Medicine, Jilin Medical University, Jilin, China; ^2^ Medical Image Center, Jilin Central Hospital, Jilin, China; ^3^ School of Basic Medical Sciences, Jilin Medical University, Jilin, China; ^4^ School of Public Health, Jilin Medical University, Jilin, China; ^5^ Department of Gynecology, Affiliated Hospital of Jilin Medical University, Jilin, China; ^6^ Department of Biomedical Sciences, Cedars-Sinai Medical Center, Los Angeles, CA, United States; ^7^ Center for Human Genetics and Genomics, New York University Grossman School of Medicine, New York, NY, United States

**Keywords:** cervical cancer, HPV, Hippo signaling pathway, YAP, TAZ, therapeutic target

## Abstract

Cervical cancer (CC) remains a major global health threat to women, with persistent infection by high-risk human papillomavirus (HPV) being the primary etiological factor. In recent years, the Hippo signaling pathway has emerged as a critical regulator of CC pathogenesis and a promising therapeutic target. Aberrant activation of its key effectors, Yes-associated protein (YAP, also referred to as YAP1) and transcriptional coactivator with PDZ-binding motif (TAZ), is closely linked to enhanced proliferation, migration, and invasion of CC cells. This review provides a comprehensive analysis of the intricate crosstalk between the Hippo pathway and HPV-driven oncogenesis. We detail specific mechanisms, such as how HPV oncoproteins (e.g., E6/E7) directly stabilize YAP/TAZ and disrupt the tumor-suppressive YAP1-LATS2 feedback loop, thereby synergistically promoting carcinogenesis. Furthermore, we explore the regulatory network involving non-coding RNAs (ncRNAs), including how miRNAs and lncRNAs modulate Hippo components to influence CC progression. Beyond mechanistic insights, this review critically evaluates the therapeutic potential of targeting the Hippo pathway, discussing innovative strategies such as small-molecule inhibitors, rational combinations with immunotherapy or chemo/radiotherapy, and the pathway’s significant role in mediating drug resistance. Ultimately, this work aims to consolidate a theoretical foundation for developing novel, mechanism-based treatment strategies for CC, offering new perspectives and actionable targets for future clinical intervention.

## 1 Introduction

Cervical cancer (CC), a malignant tumor of the female reproductive tract, ranks as the fourth leading cause of cancer-related mortality among women worldwide (1). Global estimates from 2022 reported approximately 660,000 new cases and 350,000 deaths attributable to CC (1). Although the widespread adoption of cervical cytology screening and human papillomavirus (HPV) vaccination has substantially reduced the incidence and mortality of CC (2), the disease continues to pose a major threat to global public health. Significant disparities remain in vaccine coverage and screening accessibility, particularly in low- and middle-income countries (3), where about 85% of CC-related deaths occur and mortality rates are 18 times higher than those in high-income nations (4). These inequalities underscore the urgent need to deepen our understanding of the molecular mechanisms driving cervical carcinogenesis and to develop novel treatment strategies, especially those applicable in resource-limited settings.

HPV is established as a necessary but insufficient cause in the pathogenesis of CC (5). The International Agency for Research on Cancer (IARC) has identified 12 high-risk HPV (HR-HPV) types as Group 1 carcinogens (6), among which persistent infection is the most common precursors to CC development (7). Such persistent can lead to high-grade cervical intraepithelial neoplasia (CIN), including CIN2 and CIN3. It is estimated that nearly 30% of CIN3 lesions progress to invasive CC over 30 years (8). Timely intervention at this precancerous stage can profoundly alter the disease course. Advances in cellular and molecular biology have highlighted the central role of signaling pathway dysregulation in driving uncontrolled proliferation and invasiveness of cancer cells (9). Therefore, elucidating the molecular mechanisms of CC and developing targeted therapeutic strategies represent critical objectives in ongoing effort to combat this disease.

Beyond HR-HPV infection, genetic and epigenetic alterations in host cells significantly contribute to the malignant transformation of CC. In particular, dysregulation of the Hippo signaling pathway has emerged as a crucial oncogenic driver. This pathway regulates fundamental cellular processes such as proliferation, apoptosis, invasion, migration, tissue repair, regeneration, and epithelial-mesenchymal transition (EMT) (10, 11). In recent years, it has attracted considerable attention as a promising therapeutic target in oncology (12). Accumulating evidence emphasizes the importance of Hippo pathway dysregulation in the development and progression of multiple malignancies (8, 13, 14).

Reinforcing its relevance in CC, a comprehensive analysis of The Cancer Genome Atlas (TCGA) data revealed significant amplification of the 11q22 locus, a genetic alteration predominantly observed in advanced squamous cell carcinomas and rarely in adenocarcinomas (15). This amplification results in elevated mRNA and protein expression of Yes-associated protein (YAP, also referred to as YAP1), a key transcriptional coactivator of the Hippo pathway (15). To further validate this multi-omics effect of YAP1 gene amplification at a pan-cancer level and specifically in CC, we analyzed data from The Cancer Genome Atlas Cervical Squamous Cell Carcinoma and Endocervical Adenocarcinoma (TCGA-CESC) cohort. As shown in **Supplementary Figure 1A**, YAP1 mRNA expression showed a strong positive correlation with its protein abundance (Pearson r=0.85, p=1.24e-48), indicating that transcriptional regulation is a primary determinant of YAP1 protein levels. More importantly, samples carrying YAP1 gene amplification exhibited significantly higher YAP1 protein expression compared to diploid samples (p=0.0007) (**Supplementary Figure 1B**). These data confirm that YAP1 gene amplification drives the overexpression of functional YAP1 protein through upregulating its mRNA transcription. Moreover, multi-omics studies incorporating TCGA data have uncovered critical crosstalk between YAP activation and miR-200a/b downregulation in promoting EMT and CC progression. YAP is markedly upregulated in the EMT cluster and serves as a defining marker of this subtype. Concurrently, miR-200a-3p expression is substantially repressed, frequently due to promoter hypermethylation. Integrated miR–mRNA/protein correlation analyses indicate that the miR-200 family negatively regulates EMT-related factors including ZEB1, ZEB2, and YAP itself. The coordinated overexpression of YAP and ZEB1, coupled with miR-200a/b silencing, creates a powerful pro-EMT feedback loop that enhances tumor invasion and metastasis (15, 16). These findings establish the YAP–miR-200 axis as a central mechanistic node in EMT-mediated cervical carcinogenesis, revealing promising targets for future therapies.

The study by He et al. provides strong support for the role of YAP in CC, demonstrating moderate-to-strong YAP protein expression in 91% of tumor tissues (17). To evaluate the clinical relevance of YAP upregulation, the authors performed a pan-cancer analysis of YAP gene alterations using multidimensional genomic datasets. Notably, CC showed the highest frequency of YAP genetic alterations among all cancer types. Subsequent network analysis revealed concomitant upregulation of multiple YAP-interacting proliferation-related genes in CC cases, supporting the potential of YAP as a prognostic biomarker (17). These results robustly confirm that YAP genomic amplification and protein overexpression contribute to the initiation and progression of CC. Additionally, dysregulation of the Hippo pathway is implicated in HPV-induced carcinogenesis. The HPV E6 protein stabilizes YAP by inhibiting its proteasomal degradation, thereby establishing YAP as a downstream target of HPV in CC cells (17).

Collectively, these findings strongly implicate YAP genomic amplification in the pathogenesis and progression of CC. This review summarizes the role of Hippo pathway-associated molecules in CC and aims to provide a rationale for novel therapeutic strategies. Given that direct Hippo-targeting agents are still in early development in the context of CC, we focus on the considerable potential of targeting its key interactors and integrating these approaches with immunotherapy or chemo/radiotherapy.

## 2 Hippo pathway proteins

The Hippo signaling pathway is a highly conserved regulatory network that integrates diverse upstream cues, such as extracellular matrix (ECM) mechanics, cell polarity, cell-cell contact, and G-protein-coupled receptor (GPCR) signaling, to govern organ size, tissue homeostasis, and the balance between cell proliferation and apoptosis (18–20). At its core, the mammalian kinase cascade, comprising MST1/2 and LATS1/2, phosphorylates the transcriptional co-activators YAP and TAZ to inhibit their nuclear localization and activity (21, 22). Dysregulation of this pathway, frequently observed in cancers, results in YAP/TAZ hyperactivation, thereby driving tumorigenesis and progression (23, 24). Prior to focusing on the cancer-specific mechanisms in CC, this section first outlines the universal molecular architecture of the Hippo pathway and its common regulatory patterns across various cancers.

### 2.1 Upstream regulators of Hippo pathway

**Figure 1:**
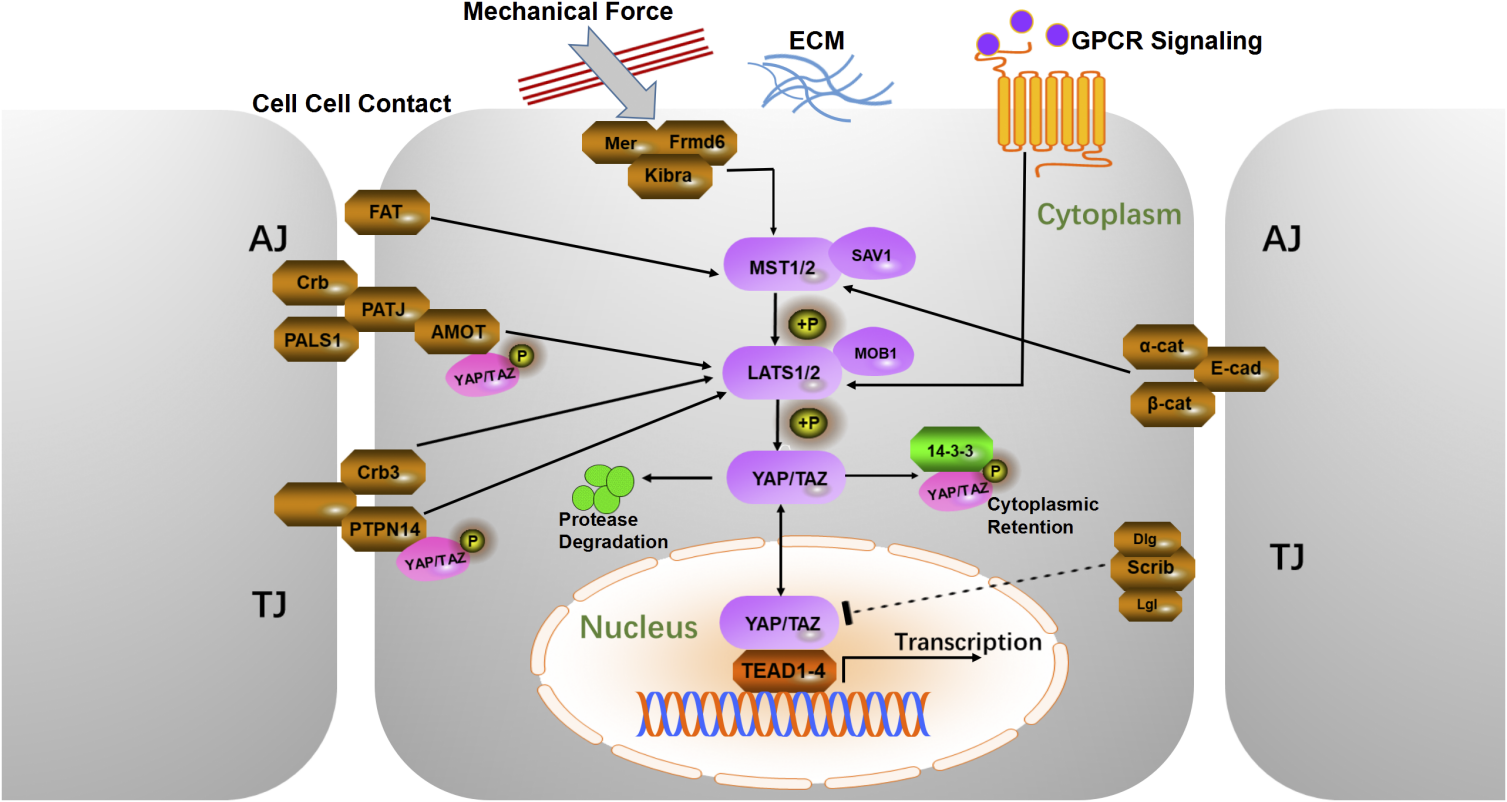
Schematic diagram of the mammalian Hippo pathway. The cells are shown with a gray outline, and the nuclei are shown in orange. Upstream regulatory proteins are in brown, intermediate core kinases are in purple, and downstream mediators are in red. Sharp arrows indicate activation, blunt arrows indicate inhibition, and dashed lines indicate unclear mechanisms. AJ, Adherens Junction; TJ, Tight Junction; α-Cat, α-Catenin; β-Cat, β-Catenin; E-cad, E-cadherin; Scrib, Scribble; Lgl, Lethal giant larvae; Dlg, Disks large protein; Amot, Angiomotin; Crb3, Crumbs3; PALS1, Protein associated with lin-7 1; PATJ, Pals1-associated tight junction protein; FAT, FAT atypical cadherin; PTPN14, Protein tyrosine phosphatase 14; Mer, Merlin; Frmd6, Ferm domain containing 6; ECM, Extracellular matrix; GPCR, G-protein coupled receptor.

Upstream regulatory factors play a crucial role in regulating various cellular processes, including cell-cell contact, ECM stiffness, cell polarity, external mechanical forces, and GPCR signaling (21) (**Figure 1**). Many of these regulators are key components of adherens junctions, tight junctions, or apical-basal polarity protein complexes, which collectively regulate the Hippo kinase cascade (20, 25). When cells come into contact, adherens junction proteins, such as Crb, PALS1, PATJ, and AMOT, bind to YAP and TAZ (WWTR1), sequestering them at the cell junctions. This sequestration prevents nuclear translocation by maintaining YAP/TAZ in their phosphorylated state, thereby inhibiting cell proliferation (21). YAP and TAZ are homologous transcriptional coactivators with overlapping functions and significant oncogenic potential (26, 27). AMOT indirectly regulates YAP activity by binding to large tumor suppressor (LATS) proteins, enhancing their kinase activity, and modulating Hippo signaling (28, 29). Crb3 further regulates YAP activity by promoting LATS-mediated phosphorylation, thereby preventing YAP nuclear translocation (30, 31). To investigate the expression pattern of CRB3 in CC, we analyzed the TCGA dataset. The results showed that CRB3 mRNA expression was significantly up-regulated in CC tissues compared to normal cervical tissues (p=0.0015) (**Supplementary Figure 2A**). Further genomic analysis revealed a positive association between CRB3 gene copy number and its mRNA expression, which reached statistical significance when comparing tumors with copy number gain to those with a diploid copy number (p=0.045; **Supplementary Figure 2B**). These findings suggest that CRB3 may act as a potential oncogene in CC, and its overexpression is partly driven by somatic copy number amplification. Among adherens junction-associated factors, E-cadherin and the α/β-catenin complex negatively regulate YAP by modulating mammalian sterile 20-like kinase (MST) activity and sequestering the YAP/14-3–3 complex in the cytoplasm (32, 33). Protein tyrosine phosphatase 14 (PTPN14) reduces nuclear YAP/TAZ levels by binding to these coactivators, promoting LATS-mediated phosphorylation, and inducing their cytoplasmic retention and ubiquitin-mediated degradation (34). Scrib, an important regulator of cell polarity and the Hippo pathway, further decreases nuclear YAP levels by initiating phosphorylation cascades at the cell membrane (35). Apical membrane-associated proteins, including the FERM domain-containing proteins Merlin (Mer) and Frmd6, interact with the WW and C2 domain-containing protein Kibra to form the Mer/Frmd6/Kibra complex (31). This complex exerts tumor-suppressive effects by recruiting and activating Hippo pathway kinases at the apical membrane (31). Mer is a well-characterized tumor suppressor, and its loss in the mouse liver leads to hepatocellular carcinoma and cholangiocarcinoma, underscoring its essential role in cellular homeostasis and tumorigenesis prevention (36).

### 2.2 FAT cadherins and Hippo pathway regulation

FAT cadherins (FAT1, FAT2, FAT3, and FAT4) are large transmembrane proteins that regulate cytoskeletal dynamics and various signaling pathways (37). Among them, FAT1 has gained significant attention due to its complex role in the Hippo signaling pathway (38). FAT1 promotes the formation of a multimeric Hippo signaling complex. This complex activates core Hippo kinases via thousand and one amino-acid protein kinases (TAOKs), leading to YAP1 inactivation (38). Notably, in cervical epithelium, multi-omics analyses have identified TAOK1 as the dominant TAOK isoform, and its expression is significantly upregulated in CC, suggesting a potentially important role for this specific kinase in the context of FAT signaling in this cancer type (39). This suggests that FAT1 is an upstream regulator of YAP1 in the Hippo pathway, a process that is often disrupted during oncogenesis. Although the functions of FAT2 and FAT3 are not fully understood, emerging evidence suggests that FAT2 regulates cell polarity and tissue architecture, which are essential for Hippo pathway activation (40). Notably, FAT3 mRNA expression patterns mirror those of FAT1 (41), and FAT3 shares functional similarities with FAT1 in its interactions with the actin cytoskeleton (41). Comparative proteomic analysis revealed a conserved C-terminal PDZ-binding motif in FAT1 and FAT3 orthologs (41), supporting the hypothesis that these proteins play complementary roles, particularly in regulating the Hippo pathway. In contrast, FAT4 is implicated in maintaining tissue integrity and may modulate Hippo signaling in a context-dependent manner (41–43). Studies have suggested that FAT2 and FAT4 may work synergistically with FAT1 to regulate the Hippo pathway, potentially coordinating with other adhesion molecules, such as the E-cadherin complex, to control the localization and activity of Hippo components (38, 41). This interplay is critical for regulating organ size and preventing tumorigenesis (38). Although current evidence points to potential functional redundancy between FAT1 and FAT3 in multiple cancer types, isoform-specific CRISPR or combinatorial siRNA experiments, especially in CC cell lines, are still lacking to quantify their individual contributions to YAP1 nuclear-cytoplasmic shuttling.

Dysregulation of FAT cadherins, particularly FAT1, is well-documented in cancer biology. Mutations in FAT1 are commonly observed in cancers, including squamous cell carcinomas of the head and neck, lungs, and cervix (38). These mutations often result in the loss of FAT1 function, leading to unchecked YAP1 activation, which promotes cell proliferation and survival and contributes to tumorigenesis (38). The molecular mechanisms by which FAT cadherins regulate the Hippo pathway are primarily mediated through direct protein-protein interactions. For instance, FAT1 interacts with the transcriptional corepressor atrophin and proteins such as HOMER-1 to HOMER-3. These proteins compete with Ena/VASP for binding to FAT1 and β-cadherin, thereby modulating the Hippo-Wnt signaling cascade (41). In contrast, FAT4 interacts with DCSH1, a ligand that may signal back to FAT4, indicating a complex and dynamic regulatory network (42, 44). Dysregulation of FAT cadherins and their effects on the Hippo pathway presents promising therapeutic opportunities. Restoring the tumor-suppressive function of FAT1 or enhancing Hippo pathway activity could offer novel strategies for treating cancers driven by YAP1 activation.

### 2.3 GPCRs and Hippo pathway regulation

**Figure 2:**
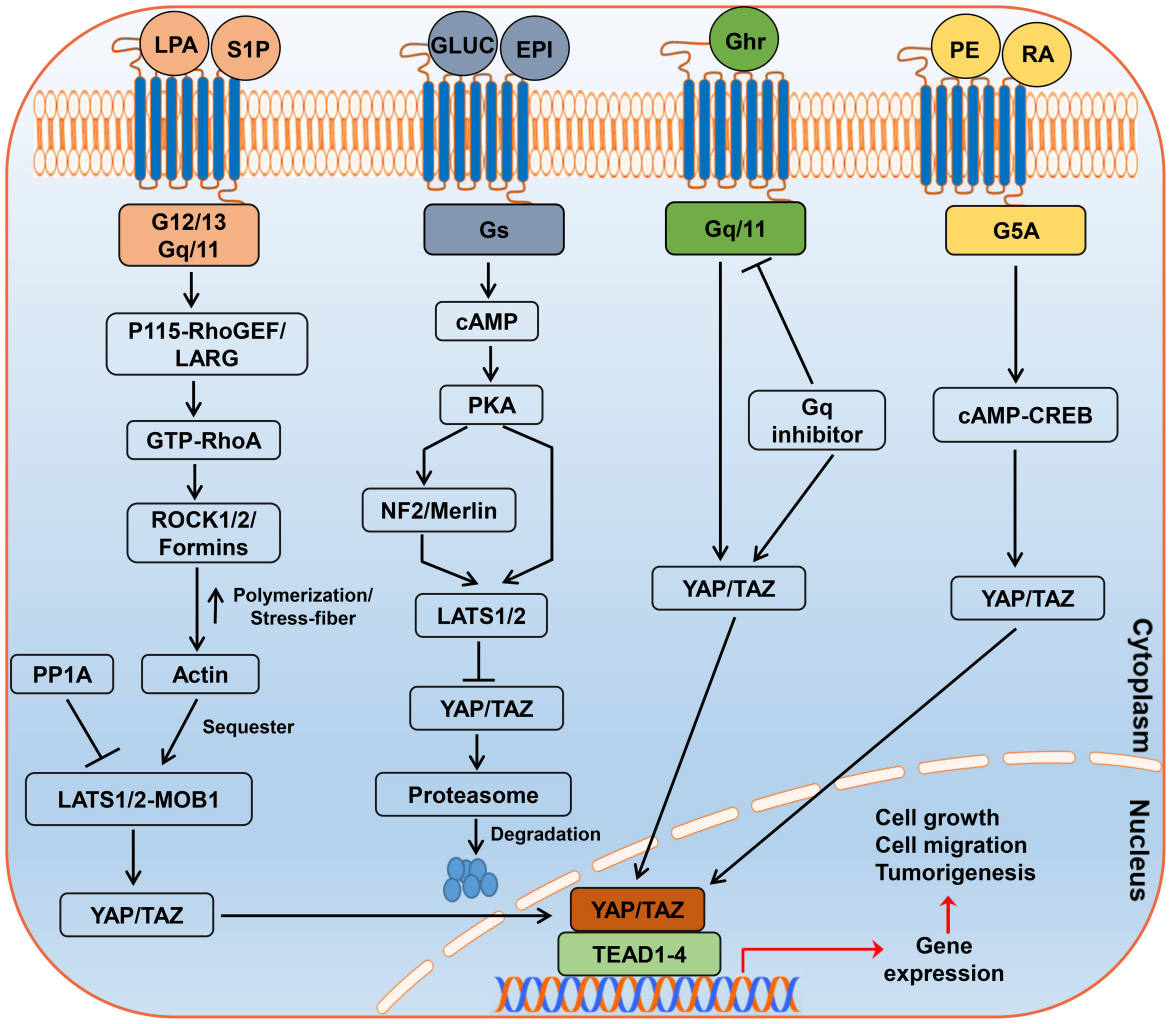
Regulation of the Hippo–YAP/TAZ Pathway by GPCRs. GPCRs regulate the activation of YAP and TAZ by initiating distinct downstream signaling cascades via different G protein subtypes. The solid arrows represent activation, whereas the blunt arrows denote inhibition. Abbreviations: LPA, lysophosphatidic acid; S1P, sphingosine 1-phosphate; GLUC, glucagon; EPI, epinephrine; Ghr, ghrelin; PE, phorbol ester; RA, retinoic acid.

GPCRs are important cell membrane signaling molecules that mediate responses to external stimuli such as hormones, chemokines, and mechanical forces (45). Upon activation, they trigger distinct downstream cascades via different G protein subtypes that regulate essential cellular processes (45) (**Figure 2**). Notably, several GPCRs modulate the Hippo pathway by controlling the activity of its core components, particularly the transcriptional coactivators YAP and TAZ (46, 47).

For example, lysophosphatidic acid (LPA) binding to LPAR1-6 (48) and sphingosine-1-phosphate (S1P) binding to S1PR1-5 (49) trigger G12/13 and Gq/11-mediated signaling, respectively. Activated G12/13 recruits Rho-specific guanine-nucleotide-exchange factors (p115-RhoGEF, LARG) that switch RhoA to its GTP-bound state (50). GTP-RhoA in turn activates Rho-kinase (ROCK1/2) and formin-family proteins, driving actin polymerization and stress-fiber formation (51, 52). These cytoskeletal changes sequester the LATS1/2–MOB1 complex at adherens junctions and promote its de-phosphorylation by protein phosphatase 1A (PP1A) (53), thereby blocking LATS1/2 kinase activity and permitting de-phosphorylated YAP/TAZ to translocate into the nucleus and activate TEAD-dependent transcription (54). Conversely, Gs-coupled receptors (e.g., glucagon or epinephrine receptors) elevate intracellular cAMP, which activates protein kinase A (PKA). PKA phosphorylates LATS1/2 directly at its (R/K)(R/K)xS/T motifs and indirectly via NF2/Merlin, enhancing LATS1/2 kinase activity and resulting in cytoplasmic retention and proteasomal degradation of YAP/TAZ (55, 56). The ghrelin receptor (GHSR) selectively activates YAP via Gq/11 independent of G12/13 signaling. Further studies have shown that this effect is reversible with a Gq inhibitor, whereas silencing G12/13 does not affect YAP activity (57). These findings suggest that modulation of the GHSR fine-tunes YAP activity, enhancing it upon activation and diminishing it when constitutive signaling is inhibited (57). Although this GHSR–G axis has been characterized only in HEK293T and colon-cancer cells to date, its relevance in CC remains to be experimentally verified.

Additionally, GPRC5A, whose expression is induced by phorbol ester and retinoic acid (58), disrupts the Hippo pathway by modulating YAP transcription through the cAMP-CREB axis, demonstrating the diversity of the GPCR-mediated regulation of YAP/TAZ in cancer progression (59). We compared GPRC5A expression levels between normal and CESC tissues. As shown in **Supplementary Figure 3A**, no significant difference in GPRC5A mRNA expression was observed between CC and normal tissues at the overall level (p=0.544). However, our further analysis revealed that its expression level is closely associated with specific genomic events. Specifically, the tumor subgroup harboring GPRC5A shallow deletion exhibited significantly lower mRNA expression compared to diploid tumors (p=0.025; **Supplementary Figure 3B**). This suggests that dysregulated GPRC5A expression is not universally present in all CCs, but rather is primarily driven by somatic copy number deletions. Finally, the involvement of G12/13, encoded by the GEP oncogene, in cancer progression has become increasingly evident (60). Elevated G12/13 expression in ovarian cancer promotes cell proliferation through YAP activation, whereas its inhibition prevents cancer cell growth (60). These findings highlight the intricate roles of GPCRs in Hippo pathway modulation and their potential as therapeutic targets for cancers characterized by the dysregulation of the Hippo pathway. The Hippo signaling pathway, which is regulated by GPCRs, is shown in **Figure 2**.

### 2.4 Kinase cascade in Hippo pathway regulation

The central mechanism of the Hippo signaling pathway involves a kinase-mediated cascade. This cascade begins when MST1/2 kinases are activated by upstream regulators assisted by Salvador homolog 1 (SAV1), a WW domain-containing protein (61). Upon activation, MST1/2 phosphorylates and activates LATS1/2 and its adaptor protein MOB kinase activator 1 (MOB1) (62). The activated LATS1/2 complex phosphorylates key residues of YAP and TAZ. Specifically, YAP has five phosphorylation sites, and TAZ has four phosphorylation sites. Phosphorylation of YAP at Ser127 and Ser381, and TAZ at Ser89 and Ser311, is closely linked to their nuclear import and degradation (63). After phosphorylation at Ser127 (YAP) and Ser89 (TAZ), both proteins interacted with 14-3–3 proteins, promoting their cytoplasmic retention. Phosphorylation of YAP Ser381 primes it for further phosphorylation by casein kinase 1 (CK1δ/ϵ) in the phosphodegron sequence, which then recruits SCFβ-TRCP E3 ubiquitin ligase, leading to YAP ubiquitination and degradation (64). Conversely, when upstream kinases are inactive, unphosphorylated YAP/TAZ translocates to the nucleus and binds to TEA domain family members (TEADs) (65), activating the expression of downstream target genes related to cell proliferation, migration, and invasion (66). A description of the mammalian Hippo pathway is presented in **Figure 1**.

The multifaceted regulators of the Hippo pathway, as detailed above, present a rich repertoire of druggable targets. Future therapeutic strategies could aim to restore tumor-suppressive inputs (e.g., by targeting FAT1-mediated signaling or GPCRs that activate LATS1/2) or directly inhibit the oncogenic YAP/TAZ-TEAD axis. The clinical success of these approaches will hinge on achieving context-specific modulation to selectively target cancer cells while minimizing on-target toxicity in healthy tissues.

## 3 Synergistic promotion of cervical epithelial carcinogenesis by HPV and the Hippo pathway

Epidemiological studies have shown that approximately 70–80% of women experience at least one HPV infection during their lifetime (67). These infections are typically transient and resolve without long-term consequences. However, a subset of women, particularly those with persistent infections, may develop precancerous cervical lesions that can ultimately progress to CC (68, 69). This progression suggests that genetic predispositions may play a significant role in the persistence of HPV infection and subsequent development of CC (70).

### 3.1 YAP1 expression in cervical lesions

YAP1 expression progressively increases across different grades of CIN, from CIN 1 to high-grade CIN 2, CIN 3, and eventually to cervical squamous cell carcinoma (CvSCC) (71). Notably, most CIN 3 and CvSCC samples are positive for either HR-HPV or YAP1 (71). Mouse model studies have shown that sustained overactivation of YAP1 in the cervical epithelium for 6–8 months is sufficient to induce invasive CC (72). These findings suggest that HPV may promote CC development by increasing YAP1 activity and expression.

### 3.2 HPV oncoproteins and YAP1 interaction in cervical carcinogenesis

**Figure 3:**
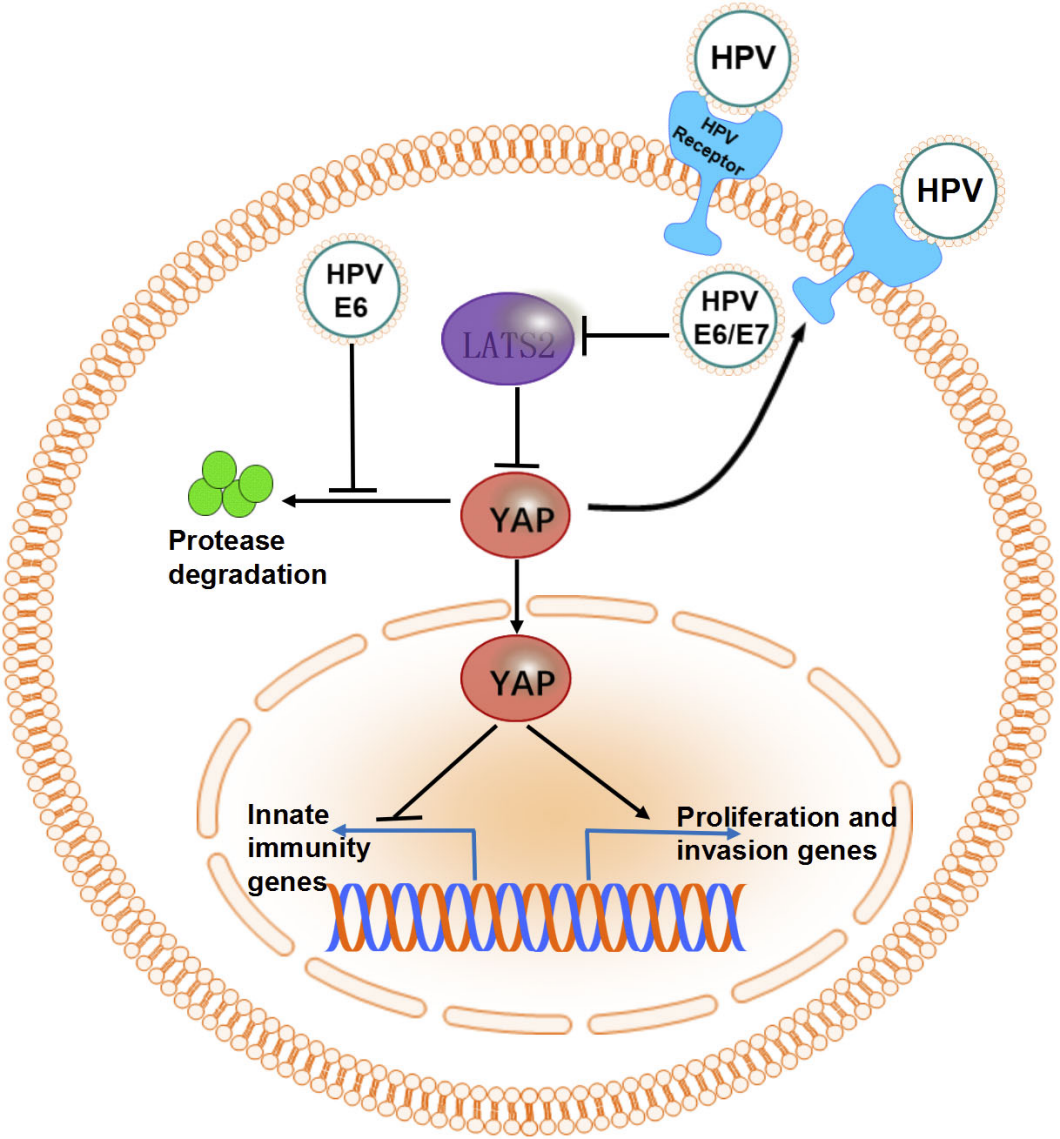
The synergistic effect of HPV and molecules in the Hippo signaling pathway. The overactivated oncogene YAP promotes the expression of HPV receptors and disrupts innate immunity by suppressing the expression of transcription factors. HR-HPV E6/E7 proteins maintain high levels of YAP in CC cells by inhibiting proteasome-mediated degradation or suppressing the expression of LATS2.

HPV16 E6/E7 oncoproteins encoded by HR-HPV are critical drivers of CC cell line proliferation (72, 73). In mouse cervical epithelial cells, co-expression of YAP1 and these oncoproteins induces invasive CC within four months (72). This progression is driven by YAP1 overactivation, which disrupts immune responses, increases HPV receptor expression, and enhances susceptibility to infection (72). Furthermore, HR-HPV E6 protein prevents YAP1 from undergoing proteasome-mediated degradation, contributing to its persistent activation (17, 74). Evidence from another study revealed that HR-HPV E6/E7 proteins, in combination with YAP1 overactivation, can induce malignant transformation in primary human cervical epithelial cells (HCvECs) (73). The interaction between HR-HPV E6/E7 and YAP1 is a crucial driver of cervical carcinogenesis. The synergistic regulation of CC progression by the Hippo signaling pathway and HPV is illustrated in **Figure 3**.

### 3.3 YAP1-LATS2 feedback in cervical homeostasis

Mechanistic investigations have revealed that YAP1 upregulates LATS2, which is essential for cervical tissue homeostasis (73). However, the expression of HR-HPV E6/E7 proteins disrupts this feedback mechanism by inhibiting LATS2 expression, thereby facilitating CC development (73). Overexpression of YAP1 has been demonstrated to promote malignant transformation in immortalized cervical epithelial cells (17) while inducing senescence in HCvECs (73). Specifically, Huang et al. provided phenotypic evidence of YAP1-induced senescence in HCvECs, showing significantly increased senescence-associated β-galactosidase (SA-β-Gal) activity (approximately 50% positive cells in HCvEC-YAP1 vs. <15% in controls), elevated formation of senescence-associated heterochromatic foci (SAHF, marked by H3K9Me3; ~50% positivity vs. <20% in controls), and upregulation of the senescence-associated secretory phenotype (SASP) marker CDKN1A (1.5-fold higher mRNA levels compared to controls) (73). Interestingly, YAP1-induced upregulation of LATS2 in HCvECs is a critical driver of this senescence process, which acts as a potent tumor-suppressive mechanism in normal cervical epithelial cells (75, 76). Genetic or environmental disruption of this feedback loop can initiate carcinogenesis in the cervical epithelium, highlighting the therapeutic potential of restoring the YAP1-LATS2 axis and its associated senescence pathways for CC prevention.

The core of this strategy lies in reactivating tumor-suppressive senescence by modulating the YAP1–LATS2 feedback loop. First, small-molecule agents can be developed to enhance LATS2 activity or inhibit YAP1, thereby promoting LATS2 phosphorylation and reinstating its tumor-suppressive function. For instance, the FDA-approved drug mitomycin C has been shown to upregulate LATS2 expression, induce cellular senescence, and suppress tumor progression in a mouse model of CC, demonstrating the feasibility of restoring this feedback loop pharmacologically (73). Second, remodeling the tumor microenvironment (TME), such as by modulating ECM stiffness, may also positively influence YAP1–LATS2 signaling. Finally, targeting upstream regulators such as PTPN14 or Crb3 can prevent their degradation and help sustain normal Hippo pathway activity (30, 34, 77). Together, these approaches offer promising clinical avenues for restoring homeostatic senescence and preventing CC development.

### 3.4 TANK-binding kinase 1 and YAP/TAZ in HPV immune evasion

**Figure 4:**
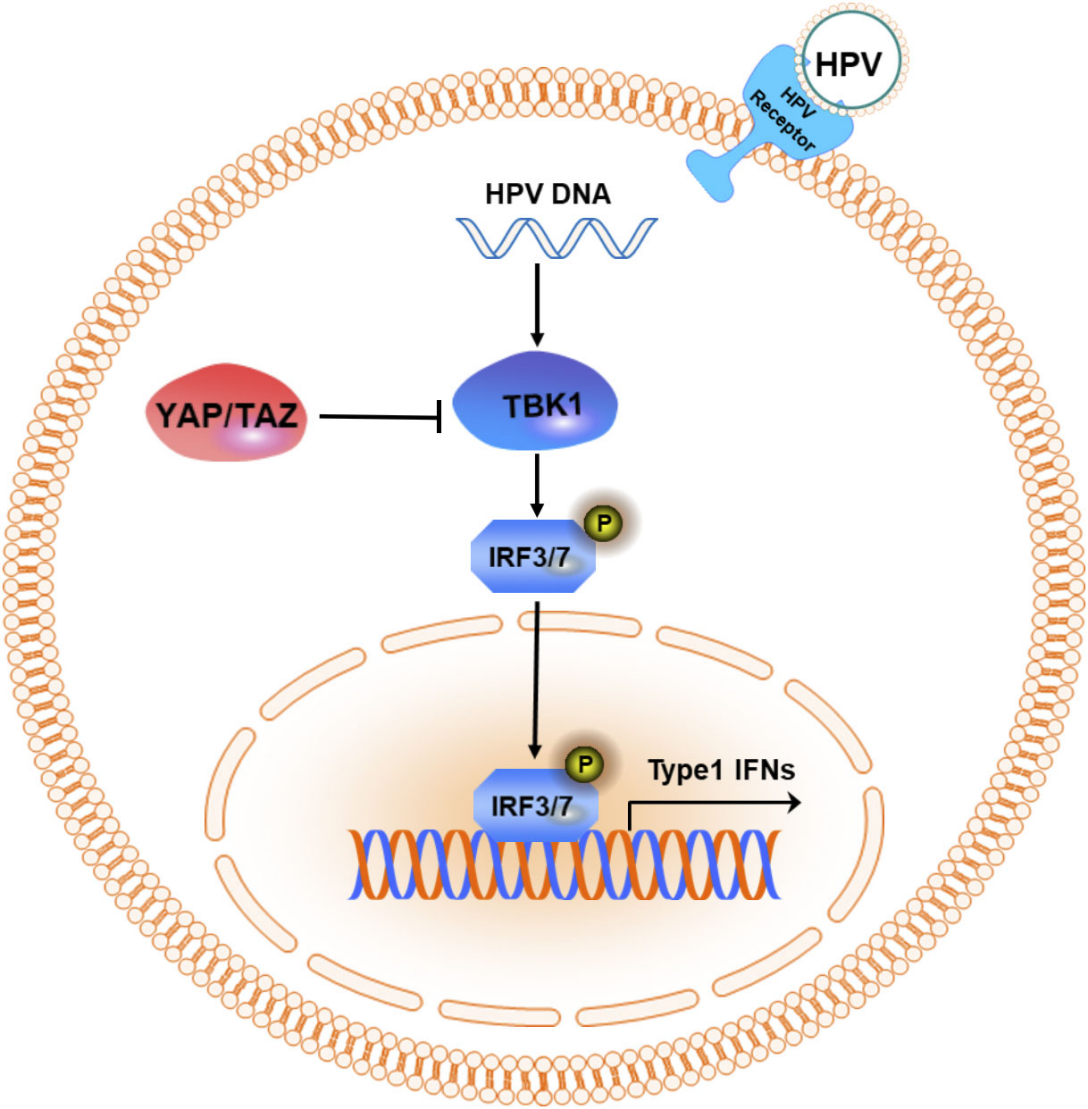
YAP/TAZ enhances the body’s susceptibility to HPV by weakening the host’s innate immunity. HPV DNA utilizes TBK1 to phosphorylate IRF3/7 and then triggers the transcription of type I IFNs. YAP/TAZ inhibits virus-induced TBK1 activation by directly binding to TBK1, thereby suppressing the host cell’s innate immune response to the virus. Abbreviations: IRF3/7, Interferon regulatory factor 3/7; IFNs, Interferons.

TANK-binding kinase 1 (TBK1), a serine/threonine kinase, plays a central role in the cytoplasmic detection of viral nucleic acids and the initiation of antiviral immune responses, acting as a critical mediator between pathogens and the host immune system (78). Upon entry into host cells, viral DNA activates TBK1, triggering the production of type I interferons, which play a pivotal role in innate antiviral immunity (79). However, YAP/TAZ proteins act as endogenous TBK1 inhibitors by directly binding to TBK1 and preventing its activation, thereby suppressing antiviral immunity and increasing susceptibility to HPV (80) (**Figure 4**). Further investigation revealed that YAP/TAZ binds to TBK1 and exerts an inhibitory effect through the C-terminal transcriptional activation domain (a.a. 291–488) of YAP. Targeting this interaction represents a novel therapeutic strategy for HPV-induced carcinogenesis. In this context, the selective TBK1 inhibitor Amlexanox, which exhibits an IC_50_ of approximately 1–2 µM in *in vitro* MBP phosphorylation assays, has attracted research interest (81). Although direct evidence regarding its potency and *in vivo* efficacy in CC models is still lacking, supportive data from other tumor types indicate its therapeutic potential. For instance, in a breast cancer bone metastasis model, Amlexanox (at around 35 mg/kg/day) combined with docetaxel significantly suppressed metastasis and prolonged survival (82). Furthermore, more potent TBK1 inhibitors such as Compound II (IC_50_≈13 nM) have shown efficacy in non-small cell lung cancer models via inhibition of the AKT pathway (83). These findings highlight the need to evaluate TBK1 inhibitors, both in CC cell lines and *in vivo* models, to assess their applicability in counteracting YAP/TAZ-mediated immune suppression and HPV-induced tumorigenesis.

In summary, the synergistic interplay between HPV infection and Hippo pathway dysregulation is a cornerstone of cervical carcinogenesis. The key mechanisms include HR-HPV E6/E7 oncoproteins directly stabilizing YAP and disrupting the tumor-suppressive YAP1-LATS2 senescence axis, thereby unlocking proliferative potential. Concurrently, YAP/TAZ activation suppresses intrinsic antiviral immunity (e.g., by inhibiting TBK1), fostering a permissive microenvironment for viral persistence and tumor development. This vicious cycle establishes a compelling rationale for dual-targeting strategies against both viral and host oncogenic drivers.

## 4 Hippo pathway in CC cell proliferation and migration

Normal cell division is essential for tissue homeostasis and organ function. During human development, a complex regulatory network controls cell proliferation. However, disruptions in these networks may lead to uncontrolled cell division, potentially driving cancer initiation (84). The Hippo signaling pathway is a fundamental regulator of this process, precisely controlling cell proliferation (18).

### 4.1 Genetic alterations in the Hippo pathway

Recurrent genetic alterations in Hippo pathway-associated genes have been observed in CC. Upstream tumor suppressors, including MST1, LATS1/2, and FAT1/2/3/4, frequently undergo deletions or mutations, whereas downstream oncogenes such as YAP1, WWTR1, and TEAD1/2/3/4 are upregulated (72). These genetic alterations are closely associated with CIN and the progression to invasive cancer (85). Notably, YAP1 overexpression in CC cells overcomes contact inhibition, sustaining proliferation. In contrast, YAP1 silencing markedly suppresses cell proliferation, induces cell cycle arrest via upregulation of p21 and p27, and impairs cancer cell migration (86). These findings identify nuclear YAP1 as a direct oncogenic target of the 11q22 amplicon, highlighting its pivotal role in CC carcinogenesis.

In terms of clinical relevance, immunohistochemical analysis has shown that YAP1 expression is significantly associated with lymph node metastasis in CvSCC (Odds Ratio [OR]=3.62, 95% Confidence Interval [CI] 1.26–10.43) and is an independent prognostic factor for overall survival (Hazard Ratio [HR]=1.21, 95% CI 1.00–2.21, p=0.048) (87). However, no significant correlation has been observed between YAP1 expression and International Federation ofGynecology and Obstetrics (FIGO) staging (I: 73.3%, II: 80.9%; χ²=0.738, p=0.39) (87). Moreover, a meta-analysis that included various cancers showed that YAP1 positivity is significantly associated with poor overall survival (HR = 1.83, 95% CI 1.47–2.28) and disease-free survival (HR = 2.11, 95% CI 1.41–3.18) (88). This supports the prognostic value of YAP1 in multiple solid tumors. However, in the context of CC, there are only a few small-scale studies that suggest a relationship between YAP1 and lymph node metastasis (87), and there is still a lack of large-scale quantitative clinical studies linking YAP1 or 11q22 amplification to FIGO stage, lymph node metastasis, and long-term patient outcomes. Future large-sample, multi-center clinical cohort studies will be an important direction for bridging mechanisms with clinical outcomes.

### 4.2 LATS1/2 tumor suppression in CC

LATS1 and LATS2 kinases are core components of the Hippo pathway and important regulators of tumorigenesis (89). Loss of LATS1/LATS2 function is frequently associated with multiple malignancies, including gastric, non-small cell lung, breast, and colorectal cancers (90–93). Immunohistochemical analysis of LATS1 expression in 80 tumor samples revealed that LATS1 was downregulated in 45% of CvSCC cases (94). Furthermore, MTT and Matrigel assays showed that LATS1 overexpression inhibits cell proliferation and invasion (94). Specifically, LATS1 exerts tumor-suppressive functions by increasing p27 levels, decreasing cyclin E and matrix metalloproteinase-9 (MMP-9), and promoting YAP phosphorylation (94). These results suggest that LATS1 acts as a tumor suppressor in CC. Activation of YAP1 or inactivation of LATS1 enhances the proliferation and invasiveness of CC cells, correlating with unfavorable prognostic factors such as low histological grade, early recurrence, and lymph node metastasis (87).

### 4.3 PTPN14 regulation of the Hippo pathway

PTPN14, an upstream regulator of the Hippo pathway, functions as a tumor suppressor (77). It forms a tumor-suppressive network with p53 and YAP, modulating Hippo signaling (95). As a cytoplasmic phosphatase, PTPN14 retains YAP in the cytoplasm in a phosphatase-independent manner (96), effectively inhibiting tumor cell proliferation and migration (21, 97). The HR-HPV E7 oncoprotein induces proteasomal degradation of PTPN14 (98, 99), promoting YAP nuclear localization and driving cellular transformation and tumorigenesis. The HPV E7–PTPN14 interaction represents a pivotal molecular event in HPV-driven carcinogenesis.

### 4.4 TAZ expression in CC

TAZ, a transcriptional coactivator structurally and functionally related to YAP, plays a critical role in multiple cancer types (100). Elevated TAZ expression has been shown to induce EMT, inhibit apoptosis, and expand the cancer stem cell population *in vitro* (101). TAZ activation promotes tumorigenesis in ovarian, breast, gastric, and oral cancers (102–105), with high TAZ levels, particularly nuclear localization, associated with poor prognosis in these cancers (100). In CC, increased TAZ expression has been observed in both tumor cells and the surrounding microenvironment (106). TAZ expression is elevated in CC tissues compared to normal tissues and promotes CD4+ T-cell infiltration into the TME (107), which is associated with diminished pathological complete response (pCR) rates (106). Analysis of the Hippo pathway in 308 CC patients from TCGA database revealed persistent TAZ amplification in CC cells, with high TAZ expression correlating with poor prognosis (15). Immunohistochemical analysis of TAZ in normal cervical tissue, high-grade squamous intraepithelial lesions (HSILs), and CvSCC revealed a progressive increase in TAZ expression, suggesting its role as a driver of CC progression (108). Overexpression of TAZ in CC cells promotes tumor growth and metastasis, and xenograft models have further confirmed its enhanced tumorigenic potential (107, 108). However, the molecular mechanisms underlying the role of TAZ in CC remain unclear.

It is noteworthy that beyond common CvSCC, TAZ dysregulation also plays a pivotal role in the rare variant, cervical clear cell carcinoma (cCCC). cCCC, a rare and aggressive form of HPV-negative CC (109, 110), is characterized by frequent mutations in WWTR1, the gene encoding TAZ. The WWTR1 S89W mutation, commonly observed in cCCC, reduces the binding of TAZ to 14-3–3 proteins, promotes TAZ nuclear translocation, and inhibits the Hippo pathway (111). Expression of the WWTR1 S89W mutant leads to enhanced tumorigenesis *in vivo*, an effect that can be reversed by targeting the TAZ/YAP1 complex with verteporfin (111). Furthermore, xenografts expressing the WWTR1 S89W mutant exhibited a highly invasive and less differentiated phenotype compared to untransformed controls (111). These findings support the hypothesis that disrupted Hippo signaling may drive cCCC rather than merely serving as a downstream consequence of earlier mutations.

## 5 Other pathways involved in CC regulation alongside Hippo

### 5.1 YAP-AREG-EGFR feedback in CC

**Figure 5:**
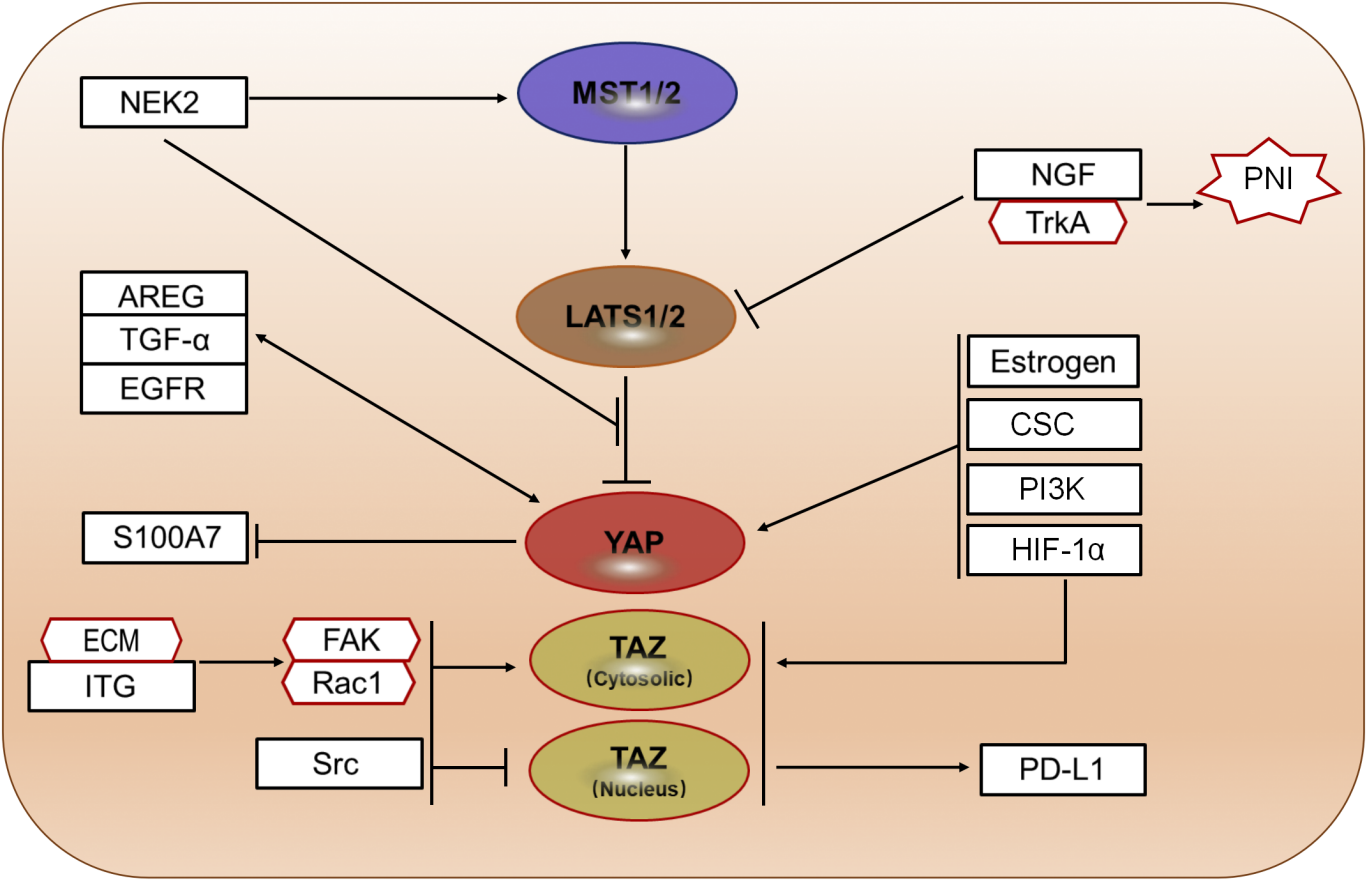
Hippo pathway molecules collaborate with other pathway proteins in the regulation of CC. The Hippo/YAP signaling pathway interacts with various intracellular pathways, including those mediated by Integrin (ITG), to participate in the occurrence and development of CC. NEK2, Never In Mitosis A (NIMA)-related kinase 2; AREG, amphiregulin; TGF-α, Transforming growth factor alpha; S100A7, S100 calcium-binding protein A7; ECM, Extracellular matrix; FAK, Focal adhesion kinase; Rac1, Ras-related C3 botulinum toxin substrate; Src, Src proto-oncogene, non-receptor tyrosine kinase; NGF, Nerve growth factor; TrkA, Tropomyosin receptor kinase A; PNI, Perineural invasion; CSC, Cigarette smoke condensate; PI3K, Phosphatidylinositol-3-kinase; HIF-1α, Hypoxia-inducible factor-1α; PD-L1, Programmed death ligand-1.

A growing body of evidence suggests that the Hippo signaling pathway interacts with multiple intracellular pathways in a complex manner and plays a central role in regulating cancer cell proliferation (17) (**Figure 5**). Amphiregulin (AREG), a protein subjected to bidirectional regulation, is a downstream target of YAP. In CC cell cultures, YAP activation markedly upregulates AREG expression, whereas YAP knockout significantly reduces AREG levels (17). Furthermore, YAP activation enhances the expression of transforming growth factor-alpha (TGF-α) and epidermal growth factor receptor (EGFR). Depletion of EGFR attenuates YAP-induced cell proliferation and AREG secretion, highlighting the importance of EGFR in this regulatory network (17).

TGF-α and AREG suppress the Hippo pathway through EGFR activation, facilitating YAP nuclear translocation and enhancing CC cell proliferation and migration. This cascade forms a positive feedback loop in which activated YAP further upregulates TGF-α, AREG, and EGFR, thereby driving CC cell malignancy (17). In a three-dimensional culture system that mimics the TME knockdown of LATS1/2 was found to activate YAP and significantly promote CC cell proliferation as well as AREG secretion, indicating that the Hippo pathway retains its oncogenic function in a context more closely resembling *in vivo* conditions (17). Furthermore, combined inhibition of YAP and EGFR signaling demonstrated a synergistic anti-tumor effect in this model (17), thereby providing a theoretical basis for subsequent validation using patient-derived xenograft models.

### 5.2 Src and integrin signaling in CC

Src, a non-receptor tyrosine kinase, plays a critical role in regulating tumor cell adhesion, migration, and proliferation through various cellular signaling pathways (112). In CvSCC, inhibition of Src family kinases in the ME180 cell line has been shown to reduce cytoplasmic TAZ levels while increasing nuclear TAZ levels, establishing a direct link between Src signaling and TAZ subcellular localization (113). This finding highlights the importance of Src in modulating the Hippo pathway effector TAZ during CC progression.

Integrins (ITGs), transmembrane receptors that mediate cell adhesion to the ECM, are central to tumor cell adhesion, migration, and proliferation (114). Their intracellular domains interact with the cytoskeleton and recruit focal adhesion kinase (FAK) upon ECM ligand binding, which in turn activates Src kinases and downstream effectors such as Rac1 (115). Elevated expression of β1 integrin is associated with advanced clinical staging and increased malignancy in various cancers, including CvSCC (116). Notably, TAZ knockdown upregulates both β1 integrin and Src expression in normal and CC cells, suggesting a synergistic interaction between the ITG-Src axis and the Hippo pathway in cervical tumorigenesis (113). These findings underscore the complex interplay between integrin signaling, Src activation, and Hippo pathway regulation in CC progression, offering potential therapeutic targets for intervention.

Additionally, Src has been demonstrated to regulate the nuclear accumulation of YAP1 under heat shock stress. In HeLa CC cells, Src serves as the primary driver of heat shock-induced YAP1 nuclear translocation. Upon temperature elevation to 42°C, Src is rapidly activated and facilitates the co-aggregation of LATS2 with itself and the protein phosphatase PP1A in the cytoplasm, forming reversible “inactivation condensates”. These structures physically sequester LATS2, which normally phosphorylates YAP1, and promote its dephosphorylation, thereby inactivating its kinase function. As a result, YAP1 escapes phosphorylation at Ser127 and is released from cytoplasmic retention. This enables rapid nuclear translocation of YAP1 within a short timeframe, initiating subsequent transcriptional programs. The entire process, from Src activation to condensate formation, LATS2 inactivation, and ultimately YAP1 nuclear entry, can be completely blocked by Src inhibitors, confirming the causal sequence of this regulatory axis (117).

### 5.3 Epigenetics and Hippo pathway in CC

DNA methylation and hydroxymethylation are fundamental epigenetic mechanisms that play pivotal roles in CC initiation and progression (118). Aberrant epigenetic modifications, particularly cytosine methylation (5-mC) and hydroxymethylation (5-hmC), can dysregulate gene expression and drive tumorigenesis (119). Epigenomic analyses comparing normal cervical epithelium, CIN, and CC samples have revealed a global decline in methylation and hydroxymethylation during cancer progression. These studies have identified specific genomic loci enriched in Hippo pathway-related genes that undergo such alterations (120). Further corroboration comes from studies on DNA methylation patterns in HPV-infected tissues, highlighting the role of epigenetic changes in CC development (121). These modifications not only accelerate cancer progression but also represent potential prognostic markers (120). The enrichment of these modifications in Hippo pathway-related genes suggests a mechanistic link between epigenetic dysregulation and Hippo pathway activation in CC. These findings underscore the importance of epigenetic mechanisms in CC and highlight their potential as therapeutic targets and biomarkers for disease progression.

Beyond DNA-level modifications, RNA methylation, notably N6-methyladenosine (m6A), also plays a critical role in post-transcriptional regulation (122). In CC, the RNA methyltransferase METTL3 is frequently overexpressed and accelerates the degradation of target mRNAs through m6A modification, thereby remodeling tumor-related transcriptional networks (122). Meanwhile, Discs large homolog 2 (DLG2), a member of the membrane-associated guanylate kinase family that regulates cell polarity and interacts with the Hippo signaling pathway, has been implicated as a potential tumor suppressor (123, 124). A recent study integrating clinical samples, cellular models, and xenograft mouse experiments revealed that METTL3 directly targets DLG2 mRNA via m6A modification, significantly shortening its half-life and leading to consistently low DLG2 expression in CC tissues and cell lines (122).

Elevation of DLG2 levels, achieved either through METTL3 inhibition or exogenous overexpression, promoted LATS1 expression and enhanced YAP/TAZ phosphorylation, thereby reactivating the Hippo pathway. This resulted in suppressed transcriptional co-activation by YAP/TAZ, significantly reduced cell proliferation, migration, and invasion, increased apoptosis, and markedly inhibited tumor growth *in vivo*. Conversely, simultaneous knockdown of DLG2 almost completely abolished the METTL3 depletion-induced reactivation of the Hippo pathway and its antitumor effects (122). These findings demonstrate that the METTL3–DLG2–Hippo/YAP axis is a critical regulatory cascade driving malignant progression in CC, providing a novel theoretical basis and potential therapeutic strategies for targeting METTL3 or activating DLG2 to restore the tumor-suppressive function of the Hippo pathway.

### 5.4 Nerve growth factor/TrkA and Hippo pathway in CC

Nerve growth factor (NGF), a neurotrophic factor, binds to specific receptors such as TrkA (a tyrosine kinase) and regulates cell proliferation, survival, and apoptosis (125). Emerging studies highlight perineural invasion (PNI) as a key factor in malignant tumor progression, contributing to local recurrence and reduced postoperative survival (126, 127). In CC, PNI is associated with a poor prognosis (128, 129). Compared to normal cervical tissue, NGF and TrkA are significantly overexpressed in CC tissues, with PNI detected in 27% of TrkA-positive tumors (130). This overexpression of NGF and TrkA facilitates PNI (131) and is closely linked to lymphatic invasion, a major risk factor for CC recurrence and poor survival (132). Thus, NGF and TrkA expression may serve as valuable prognostic biomarkers for CC (131).

Mechanistically, NGF promotes CC progression by modulating cellular signaling pathways, particularly the Hippo pathway. NGF inhibits the Hippo pathway by inactivating LATS1, leading to YAP activation and subsequent enhancement of CC cell proliferation and migration (133). This regulatory mechanism underscores the critical role of the NGF-Hippo-YAP axis in CC progression and highlights its potential as a therapeutic target. Notably, NGF inhibitors, currently in clinical trials for pain treatment (134), could be repurposed to target the Hippo pathway in CC, offering a promising strategy for improving patient outcomes.

### 5.5 NEK2 modulation of YAP in CC

NEK2, a conserved regulator of cell division, plays a critical role in various cancers (135–137). Localized in centrioles, NEK2 facilitates their separation and regulates spindle formation, ensuring proper cell cycle progression (135). Aberrant NEK2 expression disrupts these processes, leading to aneuploidy and chromosomal instability during cell division (138). NEK2 is overexpressed in multiple cancers, including breast, ovarian, prostate, cervical cancer, and leukemia (139). In CC, NEK2 upregulation is associated with lymph node metastasis, advanced tumor stage, and poor prognosis (140–142). Notably, targeting NEK2 has been shown to inhibit cervical tumorigenesis and enhance tumor sensitivity to radiotherapy, highlighting its potential as a therapeutic target (140).

Mechanistically, NEK2 drives tumor progression by modulating the Hippo pathway. It promotes YAP nuclear accumulation through cytoplasmic dephosphorylation at Ser127, thereby enhancing YAP activity (143). Additionally, NEK2 inactivates the Hippo pathway by dephosphorylating MST1/2, further increasing YAP levels and contributing to tumor progression (143). These findings underscore the central role of NEK2 in CC progression and suggest that targeting NEK2 could provide a dual benefit by inhibiting tumor growth and enhancing the efficacy of existing therapies.

### 5.6 Hypoxia-induced YAP/TAZ in CC

Hypoxia, a hallmark of solid tumors, is closely associated with poor clinical outcomes in various cancers (144–146). Adaptation to oxygen fluctuations is critical for tumor progression, and hypoxia-inducible factor-1α (HIF-1α) serves as a key transcription factor mediating cellular responses to hypoxia (147). HIF-1α promotes angiogenesis and regulates genes essential for cell survival, metabolism, and drug resistance, making it a significant therapeutic target (148–150). It activates over 100 genes involved in abnormal proliferation, metabolic reprogramming, invasion, metastasis, and therapy resistance, with elevated HIF-1α expression strongly correlated with tumor initiation, progression, and poor prognosis (150–152).

In CvSCC, HIF-1α, YAP, and TAZ are highly expressed compared to normal cervical and CIN tissues (153). Mechanistically, HIF-1α drives CvSCC cell proliferation, invasion, and migration by activating YAP/TAZ downstream of the Hippo pathway (153). Additionally, upstream regulators of HIF-1α, such as estrogen, cigarette smoke condensate, and PI3K hyperactivation, enhance YAP1 activity in HCvECs, further promoting cervical tumorigenesis (74).

To bridge the gap between *in vitro* and *in vivo* findings and further validate the role of HIF-1α in tumor cell proliferation under physiological conditions, the authors injected HIF-1α-overexpressing C33a cells and HIF-1α-knockout SiHa cells into nude mice. Tumors derived from HIF-1α-overexpressing C33a cells were significantly larger than those in control mice, whereas tumors from HIF-1α-knockout SiHa cells were markedly smaller. In the HIF-1α overexpression group, increases in tumor volume and weight were accompanied by elevated YAP/TAZ expression within the tumors; conversely, the opposite effects were observed following HIF-1α inhibition (153). These results underscore a functional interplay between hypoxia, HIF-1α, and Hippo pathway activation in CC progression, and suggest promising therapeutic strategies targeting this axis.

### 5.7 Programmed cell death protein-1/programmed death-ligand 1 in CC immune evasion

Programmed cell death protein-1 (PD-1) and its ligand Programmed Death-Ligand 1 (PD-L1) are immunoregulatory proteins that play a critical role in tumor immune evasion (154). The interaction between the Hippo pathway and PD-1/PD-L1 has emerged as a key driver of immune evasion in various cancers, including CC (107, 155). Within the Hippo pathway, MST1/2 and LATS1/2 act as tumor suppressors by inhibiting PD-L1 expression, whereas the downstream effectors YAP and TAZ promote PD-L1 upregulation, thereby impairing T-cell function and facilitating immune escape (155). In CC, elevated TAZ levels are strongly correlated with increased PD-L1 expression, driving tumor proliferation, metastasis, and resistance to apoptosis (107). This TAZ-driven PD-L1 modulation plays a pivotal role in CC progression by enabling tumors to evade immune surveillance.

Clinical studies have demonstrated significantly elevated expression of both TAZ and PD-L1 in CC tissues across different stages, subtypes, and age groups compared to normal cervical tissue (107). This upregulation is associated with enhanced immune cell infiltration and suppression within the TME. Furthermore, in lung cancer cells, the interaction between TAZ and TEAD has been shown to be essential for PD-L1 activation (156), suggesting a similar regulatory mechanism may operate in CC. These findings underscore the central role of the Hippo pathway in CC immune evasion and highlight the potential therapeutic value of targeting both TAZ and PD-L1 to enhance the efficacy of immunotherapy.

### 5.8 S100A7-mediated EMT in CC

S100A7, also known as psoriasin, is an EF-hand calcium-binding protein implicated in tumorigenesis and cancer progression (157–160). In CC, S100A7 is significantly upregulated compared to normal cervical tissues, with immunohistochemical analysis revealing high expression in high-grade CIN, suggesting its role in early tumor progression (161). Overexpression of S100A7 promotes migration, invasion, and metastasis in CC cells, partly through the induction of EMT (161, 162). EMT enables epithelial cells to acquire mesenchymal properties, as evidenced by increased expression of mesenchymal markers (N-cadherin, vimentin, and fibronectin) and decreased expression of the epithelial marker E-cadherin (161). These changes enhance cell migration and invasion, underscoring the critical role of S100A7 in CC progression.

Furthermore, the relationship between S100A7 and the Hippo pathway effector YAP has been explored in CvSCC. In well-differentiated CvSCC cells, S100A7 expression positively correlates with phosphorylated YAP (pYAP-S127) but inversely with nuclear YAP, suggesting that nuclear YAP inhibits S100A7 expression (163). Mechanistically, TEAD1 mediates the YAP-induced transcriptional repression of S100A7. Notably, S100A7 expression is low and weakly inducible in poorly differentiated CC cells, highlighting the context-dependent regulation of S100A7 by YAP (163). These findings underscore the importance of YAP-mediated regulation of S100A7 in CC progression and provide insights into potential therapeutic targets. The regulatory proteins of the Hippo pathway and their impact on the progression of CC are summarized in **Supplementary Table 1**.

Collectively, this section underscores that the oncogenic power of the Hippo pathway in CC is amplified through its extensive integration with diverse signaling networks. Cross-talk with the EGFR, Src/Integrin, NGF/TrkA, HIF-1α, and PD-L1/PD-1 axes, as well as epigenetic regulators like METTL3, demonstrates that YAP/TAZ serve as central hubs coordinating proliferation, metastasis, immune evasion, and therapy resistance. This context-dependent regulation implies that therapeutic efficacy will likely hinge on understanding and targeting these critical pathway interactions.

## 6 The clinical potential of targeting Hippo pathway-interacting molecules in CC

### 6.1 EGFR inhibitors

Although molecules interacting with the Hippo pathway theoretically influence CC through this mechanism, very few have progressed to clinical trials. Cetuximab, a chimeric monoclonal antibody targeting EGFR, inhibits ligand binding and tyrosine kinase activation (164–167). In an initial phase II monotherapy trial (Santin et al., n=35), heavily pretreated patients received cetuximab (400 mg/m² loading dose, then 250 mg/m² weekly until progression or intolerance). No objective responses were observed (objective response rate [ORR], 0%); however, 5 patients (14.3%) remained progression-free at 6 months, and median overall survival (OS) was 6.7 months. Grade ≥3 toxicities, primarily rash, fatigue, and gastrointestinal events, were manageable. There were no grade 4 events or treatment-related deaths (168).

A subsequent phase II trial evaluated cetuximab combined with cisplatin (Farley et al., n=69). Patients received the same cetuximab regimen plus cisplatin (30 mg/m² on days 1 and 8 of a 21-day cycle). Among 69 evaluable patients, 8 achieved partial responses (ORR 11.6%). Kaplan–Meier estimates showed a 6-month progression-free survival (PFS) rate of 15–20% and median OS of 8.77 months (95% CI 7.56–10.09). Comparison with historical cisplatin controls from the Gynecologic Oncology Group (GOG) trials 169/179 yielded hazard ratios near 1, indicating no synergistic benefit. Grade ≥3 adverse events included metabolic abnormalities, rash, fatigue, and gastrointestinal effects; 4 patients (5.8%) had grade 4 events. No treatment-related deaths occurred (169). Immunohistochemistry revealed 98% EGFR positivity (median cellular expression: 81%), but high EGFR expression (>81% positive cells) correlated with shorter PFS (HR 1.76), suggesting a prognostic rather than predictive role (169).

Notably, in Farley’s trial, all responders had squamous histology. Similarly, in Santin’s cohort, the 6-month PFS rate was 21% (5/24) in squamous-cell carcinoma versus 0% in non-squamous subtypes, indicating a modest but hypothesis-generating signal within squamous populations (168, 169). Despite limited clinical efficacy, cetuximab’s favorable tolerability and modest PFS benefit in squamous subgroups support further evaluation, particularly in combination with immuno-oncology agents or next-generation EGFR-directed therapies.

In contrast, erlotinib, an EGFR tyrosine kinase inhibitor (TKI), combined with cisplatin-based concurrent chemoradiation (E+CRT) demonstrated notable efficacy in locally advanced CC (170). In a phase II trial involving 41 previously untreated patients (36 evaluable), the regimen consisted of erlotinib (150 mg/day, initiated one week before radiotherapy), weekly cisplatin (40 mg/m² for 5 weeks), and radiotherapy (45 Gy in 25 fractions). The complete response rate was 94.4% (34/36). With a median follow-up of 59.3 months, the 2-year PFS and OS rates were 80.6% and 91.7%, respectively; at 3 years, these were 73.8% and 79.9%. Treatment-related toxicities were primarily grade 1–2 rash, diarrhea, and nausea. Grade 3 events included rash, diarrhea, and hematologic or vascular adverse effects. There were no treatment-related deaths or severe radiation toxicities, though three cases of late grade 3 proctitis or fistula were reported. Thus, E+CRT demonstrates compelling activity and acceptable safety in locally advanced CC, warranting further validation (170).

The contrasting outcomes between cetuximab and erlotinib highlight a key therapeutic principle: efficacy is context-dependent. Cetuximab’s lack of benefit in pretreated advanced disease diverges sharply from erlotinib’s success in previously untreated, locally advanced settings. This difference may be attributed to erlotinib’s mechanism as a radiosensitizing EGFR-TKI, its integration into a curative-intent regimen, and its use in a frontline population. Therefore, the future of EGFR inhibition in CC may lie not with monoclonal antibodies in the salvage setting, but with next-generation EGFR-TKIs strategically combined with established radical modalities such as chemoradiation.

### 6.2 PD-1 inhibitors

#### 6.2.1 Pembrolizumab

As a PD-1 inhibitor, pembrolizumab is a humanized IgG4 monoclonal antibody that acts through high-affinity binding to the PD-1 receptor on T cells. By blocking the interaction between PD-1 and its ligands PD-L1/PD-L2, it alleviates immune suppression and enhances T cell-mediated antitumor activity (171). This mechanism has demonstrated significant potential in CC treatment, as evidenced by two pivotal clinical trials: KEYNOTE-826, which established its role in recurrent/metastatic CC, and KEYNOTE-A18, which explored its use in the upfront treatment of locally advanced disease.

The KEYNOTE-826 clinical trial established pembrolizumab in combination with chemotherapy (± bevacizumab) as the first-line standard treatment for recurrent or metastatic CC. This global, multicenter, randomized, double-blind, placebo-controlled phase III study included a total of 617 patients. The final analysis indicated that in patients with persistent, recurrent, or metastatic CC who had not received prior systemic chemotherapy and were not candidates for curative treatment, both pembrolizumab in combination with paclitaxel + cisplatin/carboplatin and the addition of bevacizumab to this regimen significantly prolonged OS and PFS compared to the placebo group, and this benefit was independent of bevacizumab use (172). In the PD-L1 combined positive score (CPS) ≥1 population, consistent benefits were observed regardless of bevacizumab use, with HR indicating significant improvements: for the bevacizumab combination group, the HRs for OS and PFS were 0.60 (95% CI 0.45-0.79) and 0.56 (95% CI 0.43-0.73), respectively, while for the non-bevacizumab group, the HRs were 0.61 (95% CI 0.44-0.85) and 0.61 (95% CI 0.44-0.85). Objective response rates were significantly higher, and the duration of response was also longer. In terms of safety, in the bevacizumab-containing subgroup, the incidence of grade ≥3 treatment-related adverse events was 74.0% vs. 66.8%, and immune-related adverse events were 16.3% vs. 5.7%. In the non-bevacizumab subgroup, the respective rates were 60.4% vs. 62.1% and 9.9% vs. 4.3%. No new safety signals were observed, and the overall safety profile was manageable (172). The study concluded that for patients without contraindications, pembrolizumab + chemotherapy + bevacizumab is the preferred first-line regimen. For patients with contraindications to bevacizumab, pembrolizumab + chemotherapy also provides significant clinical benefit and should be considered a new standard of care. In conclusion, KEYNOTE-826 confirmed pembrolizumab in combination with chemotherapy (paclitaxel + platinum-based) as the cornerstone of first-line treatment for recurrent/metastatic CC, including patients who are not suitable for bevacizumab.

The KEYNOTE-A18 study evaluated pembrolizumab as upfront treatment in locally advanced CC. This randomized, double-blind Phase III trial included 1060 newly diagnosed high-risk patients. Pembrolizumab combined with chemoradiation and maintenance therapy significantly improved PFS and OS compared to placebo. At a median follow-up of 17.9 months, the 24-month PFS rate was 68% in the pembrolizumab group versus 57% in the placebo group (HR = 0.70, p=0.002) (173). After 29.9 months, the 36-month OS rate was 82.6% with pembrolizumab compared to 74.8% with placebo (HR = 0.67, p=0.004) (174). The pembrolizumab group also exhibited higher objective response rates and longer duration of response. Patient-reported outcomes (PROs) indicated no significant difference in health-related quality of life (HRQoL) between groups, with over 75% of participants maintaining stable or improved quality of life. Although immune-related adverse events were more frequent, they did not substantially impact overall quality of life, supporting a favorable efficacy–tolerability profile (175). In summary, KEYNOTE-A18 established pembrolizumab combined with chemoradiation and maintenance therapy as a new standard treatment for locally advanced CC, offering an effective, well-tolerated option that preserves quality of life.

Together, these two studies address key stages of CC management and form a comprehensive, evidence-based framework for the use of pembrolizumab, fundamentally reshaping treatment strategies for this disease.

#### 6.2.2 Cemiplimab

Cemiplimab, another PD-1 inhibitor, demonstrated significant efficacy and safety in the phase III randomized, controlled EMPOWER-Cervical 1 trial (also known as GOG-3016/ENGOT-cx9). This study enrolled 608 patients with recurrent CC who had progressed after prior platinum-based chemotherapy. Participants were randomized to receive either single-agent cemiplimab (350 mg every three weeks) or investigator’s choice of single-agent chemotherapy (pemetrexed, topotecan, irinotecan, gemcitabine, or vinorelbine). The primary endpoint, OS, showed a significant improvement with cemiplimab compared to chemotherapy: median OS was 11.7 months versus 8.5 months (HR 0.67; p < 0.00001). Benefit was consistent across subgroups, regardless of PD-L1 status. Regarding safety, the incidence of any-grade adverse events was similar between the two groups (89.7% vs. 91.7%), but the cemiplimab group had a lower incidence of grade ≥3 adverse events (45.0% vs. 53.4%) (176). Furthermore, PROs indicated that cemiplimab provided clinically meaningful and superior improvements in global health status/quality of life and physical function compared to chemotherapy, with least-squares mean differences of 8.49 points and 8.35 points, respectively (p<0.001). Meaningful improvements were also observed in key symptom domains such as role function, loss of appetite, and pain (177).

In conclusion, cemiplimab not only provided significant survival benefits and a more favorable safety profile but also meaningfully improved patient-reported quality of life and symptom burden. Based on these results, cemiplimab represents a preferred second-line immunotherapy standard for recurrent CC, regardless of PD-L1 expression status.

### 6.3 PD-L1 inhibitor

Socazolimab, a recombinant fully human anti-PD-L1 monoclonal antibody, was evaluated in a phase I clinical trial for its safety, tolerability, pharmacokinetics, and preliminary efficacy in patients with recurrent/metastatic CC who had failed or were intolerant to prior platinum-based therapy (178).

The trial initially employed a traditional 3 + 3 design for dose escalation across three dose levels (5, 10, and 15 mg/kg), enrolling 12 patients. Results demonstrated that a dose of 5 mg/kg achieved nearly 90% receptor occupancy without any dose-limiting toxicities, thus this dose was selected for the expansion phase. A total of 92 patients subsequently received Socazolimab monotherapy at 5 mg/kg every two weeks until disease progression or unacceptable toxicity. The primary endpoints were safety and maximum tolerated dose (MTD) in the escalation phase, and safety and independently reviewed ORR in the expansion phase. Among the 104 patients included in the safety analysis set, adverse events (AEs) of any cause occurred in 97% of patients, with treatment-related AEs (TRAEs) observed in 63.5%. The most common TRAEs included hypothyroidism, decreased white blood cell count, elevated hepatic enzymes, and anemia. Grade ≥3 TRAEs were reported in 8.4% of patients, with no treatment-related deaths. In the 91 patients evaluable for efficacy per independent review, the confirmed ORR was 15.4%, and the disease control rate was 49.5%. Notably, ORR was comparable between PD-L1 positive (CPS ≥1) and negative (CPS <1) subgroups, at 16.7% and 17.9%, respectively. Median PFS was 4.44 months, and median OS was 14.72 months. The median time to response was 2.0 months, and the median duration of response had not yet been reached (178).

In conclusion, by blocking the PD-1/PD-L1 pathway and potentially inducing antibody-dependent cell-mediated cytotoxicity (ADCC) via its IgG1 Fc domain, socazolimab demonstrated promising preliminary efficacy and a manageable safety profile in this study, with activity observed irrespective of PD-L1 expression status. These results support its further development as a potential second-line immunotherapy option for patients with recurrent/metastatic CC.

### 6.4 PI3Kα inhibitor

Alpelisib, as a selective PI3Kα inhibitor, was evaluated in a first-in-human study involving patients with advanced solid tumors harboring PIK3CA mutations. The trial established its maximum tolerated dose at 400 mg once daily and 150 mg twice daily. Hyperglycemia was the most frequent treatment-related adverse event (51.5%), alongside nausea, decreased appetite, diarrhea, and vomiting; importantly, hyperglycemia was manageable through dose adjustment, treatment interruption, and concomitant antidiabetic medication (e.g., metformin or insulin). Among the 134 enrolled patients, which included a cohort of 5 individuals with CC, an overall objective response rate of 6.0% was observed. Notably, 3 of the 5 CC patients achieved a partial response, demonstrating promising antitumor activity in this population (179). These findings provide early clinical evidence that alpelisib has a manageable safety profile and meaningful efficacy in pretreated PIK3CA-mutant CC, supporting its further development as a targeted therapy for this molecularly defined subgroup. The current status of clinical trials for drugs targeting the Hippo interaction pathway in CC is shown in **Supplementary Table 2**.

## 7 Role of noncoding RNAs in Hippo pathway regulation of CC

### 7.1 noncoding RNAs in cancer regulation

Approximately 2% of the human genome encodes proteins, while the majority is noncoding, with many sequences transcribed into noncoding RNAs (ncRNAs) (180). Advances in sequencing technologies have highlighted the regulatory roles of both long ncRNAs (lncRNAs, >200 nucleotides) and small ncRNAs (~20 nucleotides) (181–183). Although lncRNAs do not encode proteins (184), they regulate various cellular processes, such as cell cycle control, apoptosis, chromatin remodeling, and RNA splicing (185, 186). In cancer, lncRNAs exhibit distinct expression profiles, and their dysregulation is often linked to tumorigenesis, making them potential biomarkers and therapeutic targets (187, 188).

In contrast, microRNAs (miRNAs), the most abundant small ncRNAs, regulate gene expression post-transcriptionally by binding to mRNAs and silencing their targets (189). Most miRNAs originate from the introns of protein-coding genes and are transcribed by RNA polymerase II (190). In cancer, miRNA expression varies across tissues, indicating their role in tumor progression and regulation (191). For instance, some miRNAs act as oncogenes by promoting cell proliferation and survival, while others function as tumor suppressors by inhibiting these processes or promoting chemosensitivity (192). These findings underscore the importance of ncRNAs in cancer biology and highlight their potential as diagnostic markers and therapeutic targets.

### 7.2 lncRNAs and miRNAs in Hippo pathway regulation in CC

Recent studies have highlighted the interplay between lncRNAs and miRNAs in the regulation of the Hippo signaling pathway in CC. For instance, the oncogenic lncRNA HOTAIR promotes cancer cell migration and invasion by inhibiting YAP1 phosphorylation at Ser127 (193). HOTAIR expression correlates with tumor grade and prognosis in various cancers (194) and modulates oncogenic pathways, including those governing migration and metastasis (195, 196). Mechanistically HOTAIR recruits DNA methyltransferase 3β (DNMT3B) to repress LATS1 through methylation-mediated silencing, thereby reducing YAP1 phosphorylation at Ser127 and enabling its nuclear translocation. This enhances YAP1 transcriptional activity, promoting cancer cell motility (193).

In addition, linc00887, a long intergenic noncoding RNA, suppresses the proliferation and invasion of HeLa and C33A CC cell lines. However, miR-454-3p overexpression counteracts these effects in HeLa cells (197). Both linc00887 and miR-454-3p target FRMD6, a key regulator of the Hippo pathway, thereby modulating the FRMD6-Hippo pathway to suppress CC progression (197).

Furthermore, STK4 (MST1), a Hippo pathway regulator, is markedly downregulated in cervical disease samples and cancer cell lines compared to healthy controls (198). Reintroduction of STK4 inhibits HPV-positive cervical cell proliferation by reducing nuclear YAP localization and suppressing YAP-dependent gene expression (198). HPV E6 and E7 oncoproteins upregulate miR-18a, which targets the 3’-UTR of STK4 and maintains low STK4 levels in CC cells. miR-18a knockdown increases STK4 expression, activates the Hippo pathway, and suppresses cell proliferation. These findings reinforce the tumor-suppressive role of STK4 and highlight Hippo pathway activation as a potential therapeutic strategy for HPV-positive CC (198).

### 7.3 Extracellular vesicles-associated miRNA in CC metastasis

ncRNAs can influence CC progression through mechanisms beyond direct regulation of the Hippo pathway. Extracellular vesicles (EVs), which play a significant role in cancer pathophysiology and treatment, have been extensively studied in this context (199). Wang et al. demonstrated that EVs derived from CC cells carry miR-146a-5p, a miRNA upregulated in CC tissues (200). Elevated miR-146a-5p expression promotes metastasis by enhancing invasion, anoikis resistance, and EMT.

Mechanistically, miR-146a-5p activates YAP by targeting WWC2, a member of the WWC protein family that normally inhibits YAP transcriptional activity, cell proliferation, and organ growth (200). By suppressing WWC2, miR-146a-5p derepresses YAP, leading to enhanced cofilin phosphorylation and actin filament depolymerization. *In vivo*, EV-associated miR-146a-5p accelerates tumor metastasis through the WWC2/YAP axis, influencing actin dynamics and facilitating CC dissemination (200).

This EV-mediated mechanism of metastasis not only highlights the role of miR-146a-5p in CC progression but also suggests potential therapeutic applications. For instance, CC cells may be targeted using synthetic EVs for drug delivery, and EV-associated miR-146a-5p could serve as a circulating biomarker for diagnosis and prognosis.

## 8 Hippo signaling pathway and CC drug resistance

Current treatment modalities for CC primarily include surgery, chemotherapy, and radiotherapy. Although these approaches have improved overall survival, tumor invasion and metastasis remain major contributors to poor prognosis, particularly in patients with advanced or recurrent disease. In such cases, the one-year survival rate may drop to as low as 20% (201).

### 8.1 Chemotherapy resistance and the Hippo pathway

The AJUBA LIM protein, characterized by multiple LIM domains, has been implicated in cancer progression. Increased AJUBA expression is observed in cisplatin-resistant CC patients and is correlated with significantly shortened overall survival (202). Mechanistically, AJUBA functions as a negative regulator of the Hippo pathway (203). In CC cells, overexpression of AJUBA promotes resistance to cisplatin, enhancing cell viability and suppressing apoptosis. Conversely, knockdown of AJUBA sensitizes cells to cisplatin-induced cell death (202). This chemoresistance is mediated through the Hippo pathway effectors YAP and TAZ. AJUBA upregulates the protein expression of both YAP and TAZ, and a positive correlation exists between AJUBA and YAP/TAZ mRNA levels in patient samples (202).

Conversely, the long non-coding RNA PGM5-AS1 acts as a tumor suppressor and a cisplatin sensitizer. PGM5-AS1 expression is significantly downregulated in cisplatin-resistant CC tissues and cell lines. Its low expression is associated with higher FIGO stage, poor differentiation, and lymph node metastasis (204). Functionally, overexpression of PGM5-AS1 impairs the proliferation, migration, and invasion of cisplatin-resistant CC cells. Mechanistically, PGM5-AS1 exerts its effect by activating the Hippo pathway, as evidenced by increased phosphorylation of its core kinases MST1 and LATS1, and subsequent downregulation of the oncogenic effector YAP (204). Furthermore, PGM5-AS1 also inactivates the PI3K-AKT signaling pathway, another key driver of cell survival and chemoresistance, by reducing the levels of phosphorylated PI3K and AKT (204). This dual regulation of the Hippo and PI3K-AKT pathways highlights a complex network in which non-coding RNAs can modulate cisplatin sensitivity. Notably, silencing YAP sensitizes CC cells to cisplatin, enhances their apoptotic response, and reduces their tumorigenic potential *in vivo* (95), suggesting that combination therapies targeting AJUBA and YAP could improve treatment outcomes.

### 8.2 Radiotherapy resistance and the Hippo pathway

Radiotherapy remains the cornerstone of CC treatment; however, the development of radiation resistance often undermines its efficacy and severely affects patient outcomes (205, 206). The deubiquitinating enzyme USP21, which is overexpressed in various cancers including CC, plays a critical role in modulating protein stability and function through deubiquitination (207). Elevated USP21 expression has been observed in the tissues and cells of radiation-resistant CC patients (208). USP21 Knockout enhances the sensitivity of CC cells to radiation therapy and reduces their tumorigenic potential *in vivo* (209).

Mechanistically, USP21 promotes radiation resistance in CC by stabilizing FOXM1 via deubiquitination (209). FOXM1, a transcription factor involved in cell proliferation, interacts with YAP through TEAD1 to regulate genes essential for tumor growth. Inhibition of FOXM1 in soft tissue sarcomas has been shown to reduce tumor size *in vivo* (210), suggesting that targeting FOXM1 may offer a viable therapeutic strategy for CC. These findings highlight the potential of targeting the USP21-FOXM1-YAP axis to overcome radiation resistance and improve treatment outcomes in CC patients.

### 8.3 Therapeutic targeting of YAP in treatment resistance

Given the central role of YAP/TAZ in driving both chemoresistance and radioresistance, targeting these effectors is a promising therapeutic strategy. One promising candidate for combination therapy is Lappaol F (LAF), a natural compound extracted from burdock that exhibits potent anticancer effects in CC (211). LAF functions as a YAP inhibitor, reducing YAP levels and significantly increasing the expression of 14-3-3σ at both mRNA and protein levels (211). 14-3-3σ binds to YAP, promoting its retention and degradation in the cytoplasm (211, 212). Another well-known YAP inhibitor, verteporfin, has also been shown to reverse the tumorigenic effects driven by a constitutively active TAZ mutant (WWTR1 S89W) in cCCC (111), further validating the therapeutic potential of disrupting Hippo pathway oncogenic signaling to overcome therapy resistance.

In conclusion, hyperactivation of YAP/TAZ represents a pivotal mechanism underlying therapy resistance in CC. This is mediated through various upstream regulators (e.g., AJUBA, USP21) and non-coding RNAs (e.g., PGM5-AS1), which converge on the inhibition of the Hippo kinase cascade, leading to YAP/TAZ-driven pro-survival and anti-apoptotic programs. Importantly, pharmacological inhibition of YAP/TAZ (e.g., by Verteporfin or Lappaol F) can resensitize resistant cells, positioning the Hippo pathway as a promising therapeutic target to overcome the major clinical hurdle of treatment failure.

## 9 Discussion

CC remains a major global health threat to women, with persistent HPV infection being the primary etiological factor (7). The IARC has classified several HPV types, including HPV 16, 18, 31, 33, 35, 39, 45, 51, 52, 56, 58, and 59, as human carcinogens (6). HPV type 16 is implicated in approximately 50% of CC cases, while HPV types 16 and 18 together account for nearly 70% of CC cases (6). Progression from HPV infection to cancer typically takes 5–10 years, with an average latency of 20–25 years (213). Minor epithelial damage facilitates the entry of HPV virions into the basal layer of cervical cells, where they initiate early oncogenic lesions (214). Once internalized, HPV disrupts key signaling pathways that contribute to CC pathogenesis (215).

In recent years, the Hippo signaling pathway has emerged as a crucial regulator of cell proliferation, differentiation, and apoptosis in CC (216). The core effectors of this pathway, YAP and TAZ, are associated with increased proliferation, migration, and invasion of CC cells (216). Initially considered interchangeable, recent studies have revealed that TAZ operates independently of YAP in cancer (217). Notably, YAP is elevated in both HPV16+ and HPV18+ CC, whereas TAZ is specifically upregulated in HPV18+ cases (217). Pharmacological inhibition or shRNA-mediated knockdown of TAZ reduced the proliferation, migration, and invasion of HPV18+ CC cells, whereas YAP overexpression does not compensate for the defects observed in TAZ-depleted cells. RNA sequencing of HPV18+ cervical cells indicated that YAP and TAZ regulate distinct molecular pathways and promote carcinogenesis via independent mechanisms. This study also identified TOGARAM2 as a novel TAZ target, further clarifying its role in HPV-associated carcinogenesis (217). This functional divergence between YAP and TAZ across different HPV subtypes (summarized in **Supplementary Table 3**) underscores the potential for developing HPV type-specific targeted therapeutic strategies.

HPV drives CC development by modulating the Hippo pathway (17, 74, 217). He et al. demonstrated that constitutive activation of YAP alone in the mouse cervical epithelium is sufficient to induce CC, suggesting that HPV-mediated carcinogenesis may occur through YAP activation (72). Conversely, Huang et al. reported that YAP overactivation induces senescence in cervical epithelial cells (73). This discrepancy may stem from differences in experimental models and techniques. He et al. employed a mouse model expressing constitutively active YAPS127A under the control of doxycycline, whereas Huang et al. used retroviral expression of wild-type YAP1 or YAPS127A in HCvECs. Despite these variations, the introduction of HPV16 E6/E7 proteins into YAP-expressing cervical epithelial cells accelerated CC progression in both models (72, 73). As a core effector of the Hippo pathway, YAP plays a critical role in the invasion and metastasis of CC (218, 219). It is important to note that the oncogenic functions of YAP do not operate in isolation but engage in extensive crosstalk with other key tumor suppressor pathways, one of which is the p53 signaling network.

p53, a crucial tumor suppressor protein, primarily prevents malignant transformation by inducing cell cycle arrest, apoptosis, or DNA repair (220). In CC, the high-risk HPV E6 protein binds to and degrades p53, leading to its functional inactivation, which is a central event in driving oncogenesis (221, 222). Growing evidence has revealed multi-layered interactions between p53 and core components of the Hippo pathway. For instance, YAP can directly bind to the p53 promoter and upregulate its expression, inducing apoptosis in hepatocellular carcinoma cells, conversely, p53 can feedback to activate YAP transcription, forming a positive feedback loop (223). On the other hand, the Hippo kinase LATS1/2 can bind to and inhibit MDM2, a major negative regulator of p53, thereby blocking p53 ubiquitination and enhancing its stability (224). Conversely, in osteosarcoma, activated p53 transcriptionally upregulates LATS2, which in turn enhances the phosphorylation and inhibition of YAP, forming a negative feedback loop that amplifies anti-proliferative signals (225, 226). Based on this evidence, we hypothesize that in CC, the HPV E6 oncoprotein may simultaneously target both p53 and the Hippo pathway, disrupting their intricate interactive network to synergistically promote tumorigenesis. This regulatory mechanism awaits further experimental validation.

Another layer of complexity in the Hippo pathway’s role in cell fate decisions arises from its functional interplay with the apoptotic machinery. Specifically, a complex bidirectional regulatory relationship exists between the core Hippo kinases MST1/2 and the key apoptotic executors, Caspases (227, 228). On one hand, MST1/2 can serve as substrates for Caspases. Proteolytic cleavage relieves their autoinhibitory state, thereby activating their kinase function. In Jurkat T cells, treatment with the PI3K inhibitor GDC-0941 activates Caspase-3, -7, -8, and -9 and concurrently induces MST1 cleavage (227). Similarly, in endometrial cancer Ishikawa cells, hypoxic stress not only induces apoptosis via HIF-1α but also promotes Caspase-dependent cleavage of MST2, a process significantly inhibited by the pan-caspase inhibitor Z-VAD-FMK (228). These findings suggest that Caspase-mediated MST activation is conserved across different cell types and stress conditions. On the other hand, activated MST1/2 can positively regulate the Caspase cascade, playing a critical role in amplifying apoptotic signals. Knockdown of MST1 in Jurkat cells markedly suppresses GDC-1a941-induced activation of Caspase-3, -7, and -8, indicating that MST1 is required for the initiation of Caspase-8 and the activation of its downstream effectors (227). Notably, MST1 activation further enhances the feedback cleavage of Caspase-3/7, forming a positive feedback loop that efficiently amplifies the apoptotic signal (227). In the endometrial cancer model, the level of MST2 cleavage correlates positively with HIF-1α expression, and MST inhibition attenuates HIF-1α-mediated transcriptional activity, suggesting that MST2 may indirectly influence Caspase-dependent apoptosis by modulating HIF-1α (228).

However, as previously discussed, in CvSCC, the expression levels of HIF-1α, YAP, and TAZ are significantly elevated compared to normal cervical and CIN tissues. Mechanistically, HIF-1α drives CvSCC cell proliferation, invasion, and migration by activating YAP/TAZ downstream of the Hippo pathway (153). This observation appears to contradict the pro-apoptotic role of HIF-1α described above. The following perspectives may help reconcile this discrepancy: (1) Tissue-specific functional bias. The downstream pathways activated by HIF-1α vary significantly across tissue contexts. In endometrial cancer, HIF-1α promotes apoptosis by facilitating MST2 cleavage and activating the Caspase pathway. In CvSCC, however, it synergizes with YAP/TAZ to inhibit the tumor-suppressive function of the Hippo pathway, thereby promoting cell survival and metastasis. This functional divergence may stem from differences in upstream signaling networks and the expression profiles of transcriptional co-activators within distinct tissue microenvironments; (2) Context-dependent modes of pathway crosstalk. In endometrial cancer, the interaction between HIF-1α and MST2 may bypass the canonical YAP/TAZ axis, directly coupling to the apoptotic machinery. In CvSCC, HIF-1α may suppress the activity of upstream kinases such as MST1/2, thereby relieving the inhibition on YAP/TAZ and driving transcription programs favoring proliferation. This “pathway selection” may be governed by the expression levels and activity thresholds of the MST-LATS module in different cellular contexts; (3) Influence of stress intensity and temporal dynamics. The functional output of HIF-1α may vary with the duration and severity of hypoxia: mild or chronic hypoxia may favor adaptive proliferative responses mediated by YAP/TAZ, whereas acute or severe hypoxia tends to trigger programmed death via the MST-Caspase axis. This dynamic provides a unified framework for interpreting the contrasting “pro-survival” and “pro-death” phenotypes observed in different experimental models.

In summary, HIF-1α is not simply a pro-apoptotic or pro-proliferative factor but rather a context-dependent regulatory hub that dynamically integrates microenvironmental signals to determine cell fate. A deeper understanding of the tissue specificity and kinetic properties of the HIF-1α–MST1/2–Caspase network will provide a critical theoretical foundation for developing precision therapeutic strategies for CC.

During metastasis, cancer cells often undergo EMT to increase their invasive and migratory potentials (229). Tumor metastasis is closely associated with alterations in the TME (230, 231). The stiffness of the ECM, a key microenvironmental component, maintains the structural integrity of tumor tissues and generates signals that influence tumor cell behavior (232). Yang et al. investigated the effects of matrix stiffness on EMT and metastasis in CC and revealed that increased matrix stiffness promotes EMT and migration in HeLa cell lines both *in vitro* and *in vivo* (233). Notably, matrix stiffness regulates YAP activity through a non-Hippo mechanism involving PPIase non-mitotic interaction 1 (Pin1), which modulates YAP nuclear translocation in a phosphorylation-independent manner to support EMT in CC (233). Moreover, YAP/TAZ can coordinate the proliferation and metabolic activity of vascular endothelial cells (ECs) by upregulating MYC signaling (234). Another study demonstrated that YAP/TAZ mediates vascular endothelial growth factor (VEGF) signaling in various ECs *in vitro* and serves as a key regulator of angiogenesis (235).

As the most abundant and functionally important immune cells infiltrating the TME, tumor-associated macrophages (TAMs) mainly include M1 and M2 phenotypes. Within the TME, M2 macrophages play a critical role in tumorigenesis and progression by suppressing immune clearance, promoting cancer cell proliferation, and stimulating angiogenesis (236). Further studies revealed that YAP1 directly recruits M2 TAMs and activates tumor-initiating cells to facilitate M2 macrophage accumulation during the early stages of tumor formation (237). YAP1 expression is also associated with M2 TAM polarization. Genetic silencing or inhibition of YAP1 activity significantly reduces TAM infiltration in mouse models and downregulates the expression of key M2 polarization inducers, IL-4 and IL-13, thereby suppressing TAM polarization toward the M2 phenotype (238). These findings not only suggest potential mechanisms underlying CC metastasis, but also provide a theoretical basis for developing YAP-targeted intervention strategies.

Furthermore, YAP/TAZ play a pivotal role in mediating immunosuppression within the TME. The nuclear YAP/TAZ-TEAD complex drives the expression of chemokines CCL5 and CCL2, recruiting immunosuppressive cells such as myeloid-derived suppressor cells (MDSCs), thereby promoting immune escape and TME remodeling (239). YAP/TAZ activation also inhibits the maturation and antigen-presenting capacity of dendritic cells, while directly transcribing CXCL5. This leads to the recruitment of MDSCs into the TME via the CXCL5-CXCR2 signaling axis. The accumulated MDSCs suppress T-cell activity, further fostering an immunosuppressive TME (240). Additionally, as core effectors of anti-tumor immunity, the activation, migration, and functional states of CD4+ and CD8+ T cells are stringently regulated by the YAP/TAZ pathway. Inhibiting YAP/TAZ has been shown to reverse T-cell dysfunction and enhance the efficacy of immunotherapy (241). Collectively, these mechanisms dampen the anti-tumor immune response and compromise clinical outcomes to immunotherapy. Given the crucial role of the Hippo pathway, particularly its core effectors YAP/TAZ, in immune evasion, their aberrant activation is closely associated with tumor initiation, progression, and immune escape, highlighting their growing importance as critical therapeutic targets in cancer. For instance, metformin reduces YAP protein levels, induces YAP phosphorylation, and impedes its nuclear translocation in human colorectal cancer cells, consequently modulating PD-L1 expression (242). Verteporfin, a small-molecule inhibitor of the YAP-TEAD interaction, significantly suppresses YAP-dependent malignant phenotypes (212). However, research on immunotherapy targeting this pathway in CC remains largely unexplored. Developing multi-target inhibition strategies against YAP/TAZ may represent a promising future direction for CC immunotherapy.

Beyond immunoregulation, overcoming therapy resistance represents another critical area where YAP/TAZ-related research is poised to play a key role. Conventional therapies for CC are hindered by challenges, such as drug resistance, which contributes to high recurrence rates and poor survival in advanced-stage patients (243, 244). Studies have shown that the effectiveness of certain drugs in CC patients depends on specific genetic mutations (245). Genomic analyses from databases such as TCGA and the Catalog of Somatic Mutations in Cancer (COSMIC) revealed mutations in pathways, including RTK/PI3K/MAPK and Hippo, with PIK3CA being the most frequently altered gene (245). PIK3CA, a member of the PI3K family, is aberrantly activated and drives uncontrolled cancer cell proliferation (245). CC cell lines harboring PIK3CA mutations exhibit greater sensitivity to alpelisib than non-mutated cancer cells or normal cells. Additionally, these mutant cells demonstrated increased responsiveness to a combination of alpelisib and cisplatin (246). Further investigations revealed that alpelisib significantly attenuates the proliferation and migration of PIK3CA-mutant CC cells by inhibiting the AKT/mTOR signaling axis (246). As mentioned previously, hyperactivation of PI3K increases YAP1 activity in HCvECs, thereby inducing cervical tumorigenesis (74). Another study demonstrated that the PI3K and Hippo pathways may have a synergistic effect on the development of other diseases. The FUS-DDIT3 fusion gene, a hallmark of myxoid liposarcoma (MLS), plays a pivotal role in MLS pathogenesis. Mechanistic studies have shown that the FUS-DDIT3-driven IGF-IR/PI3K/AKT signaling cascade promotes YAP1 stability and nuclear accumulation through dysregulation of the Hippo pathway, suggesting a synergistic relationship between YAP1 and FUS-DDIT3 in MLS progression (247). These findings highlight the need to investigate whether the antitumor effects of alpelisib in CC, mediated by PI3K/AKT inhibition, are influenced by the Hippo pathway. A deeper understanding of how Hippo signaling interacts with other pathways in CC progression and drug resistance could reveal novel therapeutic strategies, both standalone and combinatorial, for patients with CC.

## 10 Future perspectives

This review has delineated the central role of the Hippo signaling pathway in HPV-driven CvSCC. Translating this robust biological understanding into clinical benefit represents the paramount challenge and opportunity for future research. Building directly upon the evidence compiled herein, we propose the following focused directions:

First, a deeper understanding of therapy resistance mechanisms is imperative. While we have highlighted the role of YAP/TAZ in mediating cisplatin resistance, their dynamic regulatory networks in radiation and immunotherapy (e.g., anti-PD-1/PD-L1) resistance remain inadequately defined. Future investigations should elucidate how the Hippo pathway engages in real-time crosstalk with other axes discussed in this review, such as PI3K-AKT, EGFR, and PD-L1, under therapeutic pressure to identify critical co-dependency nodes.

Second, concerted efforts must be made to advance Hippo-targeted strategies from concept to clinic. Given that YAP/TAZ serve as crucial downstream effectors of HPV oncoproteins, their driver role in HPV-positive CvSCC is firmly established. A highly promising avenue is the initiation of Phase I clinical trials specifically in patients with recurrent or metastatic HPV-positive CvSCC. These trials should evaluate direct TEAD inhibitors, such as VT-3989 and IAG933, which are currently under investigation in early-phase clinical trials for other solid tumors (248), in combination with standard therapies including cisplatin or radiotherapy, as well as with immune checkpoint inhibitors. Key design considerations for such trials must include patient enrichment strategies based on YAP/TAZ nuclear localization or specific genetic features such as the 11q22 amplification, alongside a proactive focus on assessing combination safety, mechanisms of resistance, and predictive biomarkers.

In conclusion, future research should be dedicated to refining our mechanistic knowledge and pioneering innovative clinical trials within the well-defined context of HPV-positive CvSCC. This dual approach promises to ultimately convert our understanding of the Hippo pathway into effective strategies that improve patient outcomes.

## Supplementary material

Supplementary Figure 1 YAP1 gene amplification drives its overexpression at the mRNA and protein levels in CC. (A) Scatter plot showing a strong positive correlation between YAP1 mRNA expression (RNA-seq RSEM) and YAP1 protein abundance (RPPA) in TCGA-CESC samples (Pearson r=0.85, p=1.24e-48). Each point represents a single tumor sample, colored by its YAP1 copy number alteration (CNA) status. **(B)** Box plot showing significantly elevated YAP1 protein expression in samples with YAP1 amplification compared to those with a diploid copy number (p=0.0007). Protein expression is presented as relative log2 expression from RPPA data. CESC, Cervical squamous cell carcinoma and endocervical adenocarcinoma; RPPA, Reverse phase protein array.

Supplementary Figure 2 CRB3 is overexpressed in CC and its expression shows a positive association with gene copy number alteration. (A) CRB3 mRNA expression is significantly up-regulated in primary CC tumors (n=305) compared to normal cervical tissues (n=3) in the TCGA-CESC cohort (p=0.0015). Expression values are shown as transcripts per million (TPM). **(B)** CRB3 mRNA expression shows a positive trend with increasing gene copy number. This trend reaches formal statistical significance when comparing tumors with CRB3 copy number gain to those with a diploid copy number (p=0.045). The elevated expression level in the single sample with CRB3 amplification (n=1) is consistent with this observed dose-response relationship. Expression values are derived from RNA-seq RSEM data.

Supplementary Figure 3 GPRC5A expression in CESC and its correlation with gene copy number alteration. (A) GPRC5A mRNA expression in normal tissues (n=3) versus CESC primary tumors (n=305) from TCGA cohort. Data are presented as log2 (RSEM value+1). **(B)** GPRC5A mRNA expression levels across different copy number alteration groups: Gain, Diploid, and Shallow Deletion. The mRNA expression data (RNA Seq V2 RSEM, log2-transformed) are shown in relation to putative copy-number alterations determined by GISTIC. A Mann-Whitney U test revealed a statistically significant decrease in GPRC5A expression in the Shallow Deletion group compared to the Diploid group (p=0.025).

## References

[1] Bray F Laversanne M Sung H Ferlay J Siegel RL Soerjomataram I. Global cancer statistics 2022: globocan estimates of incidence and mortality worldwide for 36 cancers in 185 countries. CA: Cancer J Clin. (2024) 74:229–63. doi: 10.3322/caac.21834, PMID: 38572751

[2] Bouvard V Wentzensen N Mackie A Berkhof J Brotherton J Giorgi-Rossi P. The iarc perspective on cervical cancer screening. New Engl J Med. (2021) 385:1908–18. doi: 10.1056/NEJMsr2030640, PMID: 34758259 PMC12125667

[3] Singh GK Azuine RE Siahpush M. Global inequalities in cervical cancer incidence and mortality are linked to deprivation, low socioeconomic status, and human development. Int J MCH AIDS. (2012) 1:17–30. doi: 10.21106/ijma.12, PMID: 27621956 PMC4948158

[4] Prabhu M Eckert LO. Development of world health organization (Who) recommendations for appropriate clinical trial endpoints for next-generation human papillomavirus (Hpv) vaccines. Papillomavirus Res (Amsterdam Netherlands). (2016) 2:185–9. doi: 10.1016/j.pvr.2016.10.002, PMID: 29074180 PMC5886904

[5] Subramanya D Grivas PD. Hpv and cervical cancer: updates on an established relationship. Postgraduate Med. (2008) 120:7–13. doi: 10.3810/pgm.2008.11.1928, PMID: 19020360

[6] Human Papillomaviruses. IARC monographs on the evaluation of carcinogenic risks to humans, Lyon, France: International Agency for Research on Cancer (IARC) Vol. 90. (2007). pp. 1–636 18354839 PMC4781057

[7] Walboomers JM Jacobs MV Manos MM Bosch FX Kummer JA Shah KV. Human papillomavirus is a necessary cause of invasive cervical cancer worldwide. J Pathol. (1999) 189:12–9. doi: 10.1002/(sici)1096-9896(199909)189:1<12::aid-path431>3.0.co;2-f, PMID: 10451482

[8] Mohammadi S Arefnezhad R Danaii S Yousefi M. New insights into the core hippo signaling and biological macromolecules interactions in the biology of solid tumors. BioFactors (Oxford England). (2020) 46:514–30. doi: 10.1002/biof.1634, PMID: 32445262

[9] Yang X Chen G Li W Peng C Zhu Y Yang X. Cervical cancer growth is regulated by a C-abl-plk1 signaling axis. Cancer Res. (2017) 77:1142–54. doi: 10.1158/0008-5472.can-16-1378, PMID: 27899378

[10] Akrida I Bravou V Papadaki H. The deadly cross-talk between hippo pathway and epithelial-mesenchymal transition (Emt) in cancer. Mol Biol Rep. (2022) 49:10065–76. doi: 10.1007/s11033-022-07590-z, PMID: 35604626

[11] Yu FX Zhao B Guan KL. Hippo pathway in organ size control, tissue homeostasis, and cancer. Cell. (2015) 163:811–28. doi: 10.1016/j.cell.2015.10.044, PMID: 26544935 PMC4638384

[12] Dey A Varelas X Guan KL. Targeting the hippo pathway in cancer, fibrosis, wound healing and regenerative medicine. Nat Rev Drug Discov. (2020) 19:480–94. doi: 10.1038/s41573-020-0070-z, PMID: 32555376 PMC7880238

[13] Santos-de-Frutos K Segrelles C Lorz C. Hippo pathway and yap signaling alterations in squamous cancer of the head and neck. J Clin Med. (2019) 8:2131. doi: 10.3390/jcm8122131, PMID: 31817001 PMC6947155

[14] Lo Sardo F Pulito C Sacconi A Korita E Sudol M Strano S. Yap/taz and ezh2 synergize to impair tumor suppressor activity of tgfbr2 in non-small cell lung cancer. Cancer Lett. (2021) 500:51–63. doi: 10.1016/j.canlet.2020.11.037, PMID: 33296708

[15] The Cancer Genome Atlas Research Network. Integrated genomic and molecular characterization of cervical cancer. Nature (2017) 543:378–84. doi: 10.1038/nature21386, PMID: 28112728 PMC5354998

[16] Shao DD Xue W Krall EB Bhutkar A Piccioni F Wang X. Kras and yap1 converge to regulate emt and tumor survival. Cell. (2014) 158:171–84. doi: 10.1016/j.cell.2014.06.004, PMID: 24954536 PMC4110062

[17] He C Mao D Hua G Lv X Chen X Angeletti PC. The hippo/yap pathway interacts with egfr signaling and hpv oncoproteins to regulate cervical cancer progression. EMBO Mol Med. (2015) 7:1426–49. doi: 10.15252/emmm.201404976, PMID: 26417066 PMC4644376

[18] Ma S Meng Z Chen R Guan KL. The hippo pathway: biology and pathophysiology. Annu Rev Biochem. (2019) 88:577–604. doi: 10.1146/annurev-biochem-013118-111829, PMID: 30566373

[19] Wang M Dai M Wang D Xiong W Zeng Z Guo C. The regulatory networks of the hippo signaling pathway in cancer development. J Cancer. (2021) 12:6216–30. doi: 10.7150/jca.62402, PMID: 34539895 PMC8425214

[20] Karaman R Halder G. Cell junctions in hippo signaling. Cold Spring Harbor Perspect Biol. (2018) 10:a028753. doi: 10.1101/cshperspect.a028753, PMID: 28600393 PMC5932588

[21] Meng Z Moroishi T Guan KL. Mechanisms of hippo pathway regulation. Genes Dev. (2016) 30:1–17. doi: 10.1101/gad.274027.115, PMID: 26728553 PMC4701972

[22] Yu FX Guan KL. The hippo pathway: regulators and regulations. Genes Dev. (2013) 27:355–71. doi: 10.1101/gad.210773.112, PMID: 23431053 PMC3589553

[23] Pan D. The hippo signaling pathway in development and cancer. Dev Cell. (2010) 19:491–505. doi: 10.1016/j.devcel.2010.09.011, PMID: 20951342 PMC3124840

[24] Harvey KF Zhang X Thomas DM. The hippo pathway and human cancer. Nat Rev Cancer. (2013) 13:246–57. doi: 10.1038/nrc3458, PMID: 23467301

[25] Misra JR Irvine KD. The hippo signaling network and its biological functions. Annu Rev Genet. (2018) 52:65–87. doi: 10.1146/annurev-genet-120417-031621, PMID: 30183404 PMC6322405

[26] Piccolo S Panciera T Contessotto P Cordenonsi M. Yap/taz as master regulators in cancer: modulation, function and therapeutic approaches. Nat Cancer. (2023) 4:9–26. doi: 10.1038/s43018-022-00473-z, PMID: 36564601 PMC7614914

[27] Franklin JM Wu Z Guan KL. Insights into recent findings and clinical application of yap and taz in cancer. Nat Rev Cancer. (2023) 23:512–25. doi: 10.1038/s41568-023-00579-1, PMID: 37308716

[28] Mana-Capelli S McCollum D. Angiomotins stimulate lats kinase autophosphorylation and act as scaffolds that promote hippo signaling. J Biol Chem. (2018) 293:18230–41. doi: 10.1074/jbc.RA118.004187, PMID: 30266805 PMC6254346

[29] Wang Y Yu FX. Angiomotin family proteins in the hippo signaling pathway. BioEssays: News Rev molecular Cell Dev Biol. (2024) 46:e2400076. doi: 10.1002/bies.202400076, PMID: 38760875

[30] Mao X Li P Wang Y Liang Z Liu J Li J. Crb3 regulates contact inhibition by activating the hippo pathway in mammary epithelial cells. Cell Death Dis. (2017) 8:e2546. doi: 10.1038/cddis.2016.478, PMID: 28079891 PMC5386381

[31] Ling C Zheng Y Yin F Yu J Huang J Hong Y. The apical transmembrane protein crumbs functions as a tumor suppressor that regulates hippo signaling by binding to expanded. Proc Natl Acad Sci United States America. (2010) 107:10532–7. doi: 10.1073/pnas.1004279107, PMID: 20498073 PMC2890787

[32] Quinn HM Vogel R Popp O Mertins P Lan L Messerschmidt C. Yap and B-catenin cooperate to drive oncogenesis in basal breast cancer. Cancer Res. (2021) 81:2116–27. doi: 10.1158/0008-5472.can-20-2801, PMID: 33574090

[33] Zhang C Zhu H Ren X Gao B Cheng B Liu S. Mechanics-driven nuclear localization of yap can be reversed by N-cadherin ligation in mesenchymal stem cells. Nat Commun. (2021) 12:6229. doi: 10.1038/s41467-021-26454-x, PMID: 34711824 PMC8553821

[34] Blakely WJ Hatterschide J White EA. Hpv18 E7 inhibits lats1 kinase and activates yap1 by degrading ptpn14. mBio. (2024) 15:e0181124. doi: 10.1128/mbio.01811-24, PMID: 39248565 PMC11481495

[35] Zhao D Yin Z Soellner MB Martin BR. Scribble sub-cellular localization modulates recruitment of yes1 to regulate yap1 phosphorylation. Cell Chem Biol. (2021) 28:1235–41.e5. doi: 10.1016/j.chembiol.2021.02.019, PMID: 33730553

[36] Zhang N Bai H David KK Dong J Zheng Y Cai J. The merlin/nf2 tumor suppressor functions through the yap oncoprotein to regulate tissue homeostasis in mammals. Dev Cell. (2010) 19:27–38. doi: 10.1016/j.devcel.2010.06.015, PMID: 20643348 PMC2925178

[37] Katoh M. Function and cancer genomics of fat family genes (Review). Int J Oncol. (2012) 41:1913–8. doi: 10.3892/ijo.2012.1669, PMID: 23076869 PMC3583642

[38] Martin D Degese MS Vitale-Cross L Iglesias-Bartolome R Valera JLC Wang Z. Assembly and activation of the hippo signalome by fat1 tumor suppressor. Nat Commun. (2018) 9:2372. doi: 10.1038/s41467-018-04590-1, PMID: 29985391 PMC6037762

[39] Ning L Li X Xu Y Si Y Zhao H Ren Q. Multi-omics analysis revealed that taok1 can be used as a prognostic marker and target in a variety of tumors, especially in cervical cancer. OncoTargets Ther. (2025) 18:335–53. doi: 10.2147/ott.s506582, PMID: 40109409 PMC11920640

[40] Fulford AD McNeill H. Fat/dachsous family cadherins in cell and tissue organisation. Curr Opin Cell Biol. (2020) 62:96–103. doi: 10.1016/j.ceb.2019.10.006, PMID: 31739265

[41] Zhang X Liu J Liang X Chen J Hong J Li L. History and progression of fat cadherins in health and disease. OncoTargets Ther. (2016) 9:7337–43. doi: 10.2147/ott.s111176, PMID: 27942226 PMC5138043

[42] Mao Y Francis-West P Irvine KD. Fat4/dchs1 signaling between stromal and cap mesenchyme cells influences nephrogenesis and ureteric bud branching. Dev (Cambridge England). (2015) 142:2574–85. doi: 10.1242/dev.122630, PMID: 26116666 PMC4529033

[43] Badouel C McNeill H. Snapshot: the hippo signaling pathway. Cell. (2011) 145:484–.e1. doi: 10.1016/j.cell.2011.04.009, PMID: 21529719

[44] Medina E Easa Y Lester DK Lau EK Sprinzak D Luca VC. Structure of the planar cell polarity cadherins fat4 and dachsous1. Nat Commun. (2023) 14:891. doi: 10.1038/s41467-023-36435-x, PMID: 36797229 PMC9935876

[45] Shukla AK. G protein-coupled receptors (Gpcrs). Int J Biochem Cell Biol. (2016) 77:183. doi: 10.1016/j.biocel.2016.05.008, PMID: 27179793

[46] Zhou X Wang Z Huang W Lei QY. G protein-coupled receptors: bridging the gap from the extracellular signals to the hippo pathway. Acta Biochim Biophys Sin. (2015) 47:10–5. doi: 10.1093/abbs/gmu108, PMID: 25491506

[47] Luo J Yu FX. Gpcr-hippo signaling in cancer. Cells. (2019) 8:426. doi: 10.3390/cells8050426, PMID: 31072060 PMC6563442

[48] Yung YC Stoddard NC Chun J. Lpa receptor signaling: pharmacology, physiology, and pathophysiology. J Lipid Res. (2014) 55:1192–214. doi: 10.1194/jlr.R046458, PMID: 24643338 PMC4076099

[49] Roggeri A Schepers M Tiane A Rombaut B van Veggel L Hellings N. Sphingosine-1-phosphate receptor modulators and oligodendroglial cells: beyond immunomodulation. Int J Mol Sci. (2020) 21:7537. doi: 10.3390/ijms21207537, PMID: 33066042 PMC7588977

[50] Chen Z Guo L Hadas J Gutowski S Sprang SR Sternweis PC. Activation of P115-rhogef requires direct association of Gα13 and the dbl homology domain. J Biol Chem. (2012) 287:25490–500. doi: 10.1074/jbc.M111.333716, PMID: 22661716 PMC3408165

[51] Etienne-Manneville S Hall A. Rho gtpases in cell biology. Nature. (2002) 420:629–35. doi: 10.1038/nature01148, PMID: 12478284

[52] Rossman KL Der CJ Sondek J. Gef means go: turning on rho gtpases with guanine nucleotide-exchange factors. Nat Rev Mol Cell Biol. (2005) 6:167–80. doi: 10.1038/nrm1587, PMID: 15688002

[53] Cai H Xu Y. The role of lpa and yap signaling in long-term migration of human ovarian cancer cells. Cell communication signaling: CCS. (2013) 11:31. doi: 10.1186/1478-811x-11-31, PMID: 23618389 PMC3655373

[54] Yu FX Zhao B Panupinthu N Jewell JL Lian I Wang LH. Regulation of the hippo-yap pathway by G-protein-coupled receptor signaling. Cell. (2012) 150:780–91. doi: 10.1016/j.cell.2012.06.037, PMID: 22863277 PMC3433174

[55] Yu FX Zhang Y Park HW Jewell JL Chen Q Deng Y. Protein kinase a activates the hippo pathway to modulate cell proliferation and differentiation. Genes Dev. (2013) 27:1223–32. doi: 10.1101/gad.219402.113, PMID: 23752589 PMC3690396

[56] Kim M Kim M Lee S Kuninaka S Saya H Lee H. Camp/pka signalling reinforces the lats-yap pathway to fully suppress yap in response to actin cytoskeletal changes. EMBO J. (2013) 32:1543–55. doi: 10.1038/emboj.2013.102, PMID: 23644383 PMC3671250

[57] Zindel D Mensat P Vol C Homayed Z Charrier-Savournin F Trinquet E. G protein-coupled receptors can control the hippo/yap pathway through gq signaling. FASEB journal: Off Publ Fed Am Societies Exp Biol. (2021) 35:e21668. doi: 10.1096/fj.202002159R, PMID: 34114695

[58] Iglesias González PA Valdivieso AG Santa-Coloma TA. The G protein-coupled receptor gprc5a-a phorbol ester and retinoic acid-induced orphan receptor with roles in cancer, inflammation, and immunity. Biochem Cell Biol = Biochimie biologie cellulaire. (2023) 101:465–80. doi: 10.1139/bcb-2022-0352, PMID: 37467514

[59] Fang W Yu X Deng J Yu B Xiong J Ma M. Upregulated gprc5a disrupting the hippo pathway promotes the proliferation and migration of pancreatic cancer cells via the camp-creb axis. Discover Oncol. (2023) 14:17. doi: 10.1007/s12672-023-00626-1, PMID: 36735162 PMC9898488

[60] Yagi H Asanoma K Ohgami T Ichinoe A Sonoda K Kato K. Gep oncogene promotes cell proliferation through yap activation in ovarian cancer. Oncogene. (2016) 35:4471–80. doi: 10.1038/onc.2015.505, PMID: 26804165

[61] Hansen CG Moroishi T Guan KL. Yap and taz: A nexus for hippo signaling and beyond. Trends Cell Biol. (2015) 25:499–513. doi: 10.1016/j.tcb.2015.05.002, PMID: 26045258 PMC4554827

[62] Xiong S Couzens AL Kean MJ Mao DY Guettler S Kurinov I. Regulation of protein interactions by mps one binder (Mob1) phosphorylation. Mol Cell proteomics: MCP. (2017) 16:1111–25. doi: 10.1074/mcp.M117.068130, PMID: 28373297 PMC5461541

[63] Zhao B Li L Lei Q Guan KL. The hippo-yap pathway in organ size control and tumorigenesis: an updated version. Genes Dev. (2010) 24:862–74. doi: 10.1101/gad.1909210, PMID: 20439427 PMC2861185

[64] Kim Y Jho EH. Regulation of the hippo signaling pathway by ubiquitin modification. BMB Rep. (2018) 51:143–50. doi: 10.5483/bmbrep.2018.51.3.017, PMID: 29366444 PMC5882221

[65] Zhao Y Sheldon M Sun Y Ma L. New insights into yap/taz-tead-mediated gene regulation and biological processes in cancer. Cancers. (2023) 15:5497. doi: 10.3390/cancers15235497, PMID: 38067201 PMC10705714

[66] Li HL Li QY Jin MJ Lu CF Mu ZY Xu WY. A review: hippo signaling pathway promotes tumor invasion and metastasis by regulating target gene expression. J Cancer Res Clin Oncol. (2021) 147:1569–85. doi: 10.1007/s00432-021-03604-8, PMID: 33864521 PMC11801896

[67] von Knebel Doeberitz M Reuschenbach M Schmidt D Bergeron C. Biomarkers for cervical cancer screening: the role of P16(Ink4a) to highlight transforming hpv infections. Expert Rev Proteomics. (2012) 9:149–63. doi: 10.1586/epr.12.13, PMID: 22462787

[68] Kulasingam SL Hughes JP Kiviat NB Mao C Weiss NS Kuypers JM. Evaluation of human papillomavirus testing in primary screening for cervical abnormalities: comparison of sensitivity, specificity, and frequency of referral. Jama. (2002) 288:1749–57. doi: 10.1001/jama.288.14.1749, PMID: 12365959

[69] Arbyn M Weiderpass E Bruni L de Sanjosé S Saraiya M Ferlay J. Estimates of incidence and mortality of cervical cancer in 2018: A worldwide analysis. Lancet Global Health. (2020) 8:e191–203. doi: 10.1016/s2214-109x(19)30482-6, PMID: 31812369 PMC7025157

[70] Westrich JA Warren CJ Pyeon D. Evasion of host immune defenses by human papillomavirus. Virus Res. (2017) 231:21–33. doi: 10.1016/j.virusres.2016.11.023, PMID: 27890631 PMC5325784

[71] Xiao H Wu L Zheng H Li N Wan H Liang G. Expression of yes-associated protein in cervical squamous epithelium lesions. Int J gynecological cancer: Off J Int Gynecological Cancer Soc. (2014) 24:1575–82. doi: 10.1097/igc.0000000000000259, PMID: 25304677

[72] He C Lv X Huang C Angeletti PC Hua G Dong J. A human papillomavirus-independent cervical cancer animal model reveals unconventional mechanisms of cervical carcinogenesis. Cell Rep. (2019) 26:2636–50.e5. doi: 10.1016/j.celrep.2019.02.004, PMID: 30840887 PMC6812687

[73] Huang C Lv X Chen P Liu J He C Chen L. Human papillomavirus targets the yap1-lats2 feedback loop to drive cervical cancer development. Oncogene. (2022) 41:3761–77. doi: 10.1038/s41388-022-02390-y, PMID: 35761037 PMC10399300

[74] Nishio M To Y Maehama T Aono Y Otani J Hikasa H. Endogenous yap1 activation drives immediate onset of cervical carcinoma in situ in mice. Cancer Sci. (2020) 111:3576–87. doi: 10.1111/cas.14581, PMID: 32716083 PMC7541006

[75] Campisi J. Cellular senescence as a tumor-suppressor mechanism. Trends Cell Biol. (2001) 11:S27–31. doi: 10.1016/s0962-8924(01)02151-1, PMID: 11684439

[76] Chen Z Trotman LC Shaffer D Lin HK Dotan ZA Niki M. Crucial role of P53-dependent cellular senescence in suppression of pten-deficient tumorigenesis. Nature. (2005) 436:725–30. doi: 10.1038/nature03918, PMID: 16079851 PMC1939938

[77] Wilson KE Yang N Mussell AL Zhang J. The regulatory role of kibra and ptpn14 in hippo signaling and beyond. Genes. (2016) 7:23. doi: 10.3390/genes7060023, PMID: 27240404 PMC4929422

[78] Uhlorn BL Jackson R Li S Bratton SM Van Doorslaer K Campos SK. Vesicular trafficking permits evasion of cgas/sting surveillance during initial human papillomavirus infection. PloS Pathog. (2020) 16:e1009028. doi: 10.1371/journal.ppat.1009028, PMID: 33253291 PMC7728285

[79] Basit A Cho MG Kim EY Kwon D Kang SJ Lee JH. The cgas/sting/tbk1/irf3 innate immunity pathway maintains chromosomal stability through regulation of P21 levels. Exp Mol Med. (2020) 52:643–57. doi: 10.1038/s12276-020-0416-y, PMID: 32284536 PMC7210884

[80] Zhang Q Meng F Chen S Plouffe SW Wu S Liu S. Hippo signalling governs cytosolic nucleic acid sensing through yap/taz-mediated tbk1 blockade. Nat Cell Biol. (2017) 19:362–74. doi: 10.1038/ncb3496, PMID: 28346439 PMC5398908

[81] Reilly SM Chiang SH Decker SJ Chang L Uhm M Larsen MJ. An inhibitor of the protein kinases tbk1 and ε Improves obesity-related metabolic dysfunctions in mice. Nat Med. (2013) 19:313–21. doi: 10.1038/nm.3082, PMID: 23396211 PMC3594079

[82] Bishop RT Marino S de Ridder D Allen RJ Lefley DV Sims AH. Pharmacological inhibition of the ikkϵ/tbk-1 axis potentiates the anti-tumour and anti-metastatic effects of docetaxel in mouse models of breast cancer. Cancer Lett. (2019) 450:76–87. doi: 10.1016/j.canlet.2019.02.032, PMID: 30790681

[83] Ou YH Torres M Ram R Formstecher E Roland C Cheng T. Tbk1 directly engages akt/pkb survival signaling to support oncogenic transformation. Mol Cell. (2011) 41:458–70. doi: 10.1016/j.molcel.2011.01.019, PMID: 21329883 PMC3073833

[84] Hanahan D Weinberg RA. Hallmarks of cancer: the next generation. Cell. (2011) 144:646–74. doi: 10.1016/j.cell.2011.02.013, PMID: 21376230

[85] Wang C Davis JS. At the center of cervical carcinogenesis: synergism between high-risk hpv and the hyperactivated yap1. Mol Cell Oncol. (2019) 6:e1612677. doi: 10.1080/23723556.2019.1612677, PMID: 31528691 PMC6736164

[86] Lorenzetto E Brenca M Boeri M Verri C Piccinin E Gasparini P. Yap1 acts as oncogenic target of 11q22 amplification in multiple cancer subtypes. Oncotarget. (2014) 5:2608–21. doi: 10.18632/oncotarget.1844, PMID: 24810989 PMC4058031

[87] Liu T Liu Y Gao H Meng F Yang S Lou G. Clinical significance of yes-associated protein overexpression in cervical carcinoma: the differential effects based on histotypes. Int J gynecological cancer: Off J Int Gynecological Cancer Soc. (2013) 23:735–42. doi: 10.1097/IGC.0b013e31828c8619, PMID: 23502453

[88] Sun Z Xu R Li X Ren W Ou C Wang Q. Prognostic value of yes-associated protein 1 (Yap1) in various cancers: A meta-analysis. PloS One. (2015) 10:e0135119. doi: 10.1371/journal.pone.0135119, PMID: 26263504 PMC4532485

[89] Qi S Zhu Y Liu X Li P Wang Y Zeng Y. Wwc proteins mediate lats1/2 activation by hippo kinases and imply a tumor suppression strategy. Mol Cell. (2022) 82:1850–64.e7. doi: 10.1016/j.molcel.2022.03.027, PMID: 35429439

[90] Zou J Zhou L Le Y Fang Z Zhong M Nie F. Wwp2 drives the progression of gastric cancer by facilitating the ubiquitination and degradation of lats1 protein. Cell communication signaling: CCS. (2023) 21:38. doi: 10.1186/s12964-023-01050-2, PMID: 36803368 PMC9938551

[91] Malik SA Khan MS Dar M Hussain MU Shah MA Shafi SM. Molecular alterations and expression dynamics of lats1 and lats2 genes in non-small-cell lung carcinoma. Pathol Oncol research: POR. (2018) 24:207–14. doi: 10.1007/s12253-017-0225-3, PMID: 28434174

[92] Furth N Pateras IS Rotkopf R Vlachou V Rivkin I Schmitt I. Lats1 and lats2 suppress breast cancer progression by maintaining cell identity and metabolic state. Life Sci alliance. (2018) 1:e201800171. doi: 10.26508/lsa.201800171, PMID: 30456386 PMC6238411

[93] Pan Y Tong JHM Lung RWM Kang W Kwan JSH Chak WP. Rasal2 promotes tumor progression through lats2/yap1 axis of hippo signaling pathway in colorectal cancer. Mol Cancer. (2018) 17:102. doi: 10.1186/s12943-018-0853-6, PMID: 30037330 PMC6057036

[94] Deng J Zhang W Liu S An H Tan L Ma L. Lats1 suppresses proliferation and invasion of cervical cancer. Mol Med Rep. (2017) 15:1654–60. doi: 10.3892/mmr.2017.6180, PMID: 28259899 PMC5364969

[95] Mello SS Valente LJ Raj N Seoane JA Flowers BM McClendon J. A P53 super-tumor suppressor reveals a tumor suppressive P53-ptpn14-yap axis in pancreatic cancer. Cancer Cell. (2017) 32:460–73.e6. doi: 10.1016/j.ccell.2017.09.007, PMID: 29017057 PMC5659188

[96] Michaloglou C Lehmann W Martin T Delaunay C Hueber A Barys L. The tyrosine phosphatase ptpn14 is a negative regulator of yap activity. PloS One. (2013) 8:e61916. doi: 10.1371/journal.pone.0061916, PMID: 23613971 PMC3628344

[97] Moon S Yeon Park S Woo Park H. Regulation of the hippo pathway in cancer biology. Cell Mol Life sciences: CMLS. (2018) 75:2303–19. doi: 10.1007/s00018-018-2804-1, PMID: 29602952 PMC11105795

[98] Yun HY Kim MW Lee HS Kim W Shin JH Kim H. Structural basis for recognition of the tumor suppressor protein ptpn14 by the oncoprotein E7 of human papillomavirus. PloS Biol. (2019) 17:e3000367. doi: 10.1371/journal.pbio.3000367, PMID: 31323018 PMC6668832

[99] Szalmás A Tomaić V Basukala O Massimi P Mittal S Kónya J. The ptpn14 tumor suppressor is a degradation target of human papillomavirus E7. J Virol. (2017) 91:e00057–17. doi: 10.1128/jvi.00057-17, PMID: 28100625 PMC5355602

[100] Zhou X Lei QY. Regulation of taz in cancer. Protein Cell. (2016) 7:548–61. doi: 10.1007/s13238-016-0288-z, PMID: 27412635 PMC4980330

[101] Luo J Deng L Zou H Guo Y Tong T Huang M. New insights into the ambivalent role of yap/taz in human cancers. J Exp Clin Cancer research: CR. (2023) 42:130. doi: 10.1186/s13046-023-02704-2, PMID: 37211598 PMC10201886

[102] Yang WH Huang Z Wu J Ding CC Murphy SK Chi JT. A taz-angptl4-nox2 axis regulates ferroptotic cell death and chemoresistance in epithelial ovarian cancer. Mol Cancer research: MCR. (2020) 18:79–90. doi: 10.1158/1541-7786.mcr-19-0691, PMID: 31641008 PMC6942206

[103] Díaz-Martín J López-García M Romero-Pérez L Atienza-Amores MR Pecero ML Castilla M. Nuclear taz expression associates with the triple-negative phenotype in breast cancer. Endocrine-related Cancer. (2015) 22:443–54. doi: 10.1530/erc-14-0456, PMID: 25870251

[104] Wang D Li Z Li X Yan C Yang H Zhuang T. Dub1 suppresses hippo signaling by modulating taz protein expression in gastric cancer. J Exp Clin Cancer research: CR. (2022) 41:219. doi: 10.1186/s13046-022-02410-5, PMID: 35820928 PMC9275142

[105] Li Z Wang Y Zhu Y Yuan C Wang D Zhang W. The hippo transducer taz promotes epithelial to mesenchymal transition and cancer stem cell maintenance in oral cancer. Mol Oncol. (2015) 9:1091–105. doi: 10.1016/j.molonc.2015.01.007, PMID: 25704916 PMC5528756

[106] Buglioni S Vici P Sergi D Pizzuti L Di Lauro L Antoniani B. Analysis of the hippo transducers taz and yap in cervical cancer and its microenvironment. Oncoimmunology. (2016) 5:e1160187. doi: 10.1080/2162402x.2016.1160187, PMID: 27471633 PMC4938371

[107] Han Y Liu D Li L. Increased expression of taz and associated upregulation of pd-L1 in cervical cancer. Cancer Cell Int. (2021) 21:592. doi: 10.1186/s12935-021-02287-y, PMID: 34736474 PMC8567592

[108] Chen M Zhang Y Zheng PS. Tafazzin (Taz) promotes the tumorigenicity of cervical cancer cells and inhibits apoptosis. PloS One. (2017) 12:e0177171. doi: 10.1371/journal.pone.0177171, PMID: 28489874 PMC5425199

[109] Park KJ. Cervical adenocarcinoma: integration of hpv status, pattern of invasion, morphology and molecular markers into classification. Histopathology. (2020) 76:112–27. doi: 10.1111/his.13995, PMID: 31846527

[110] Stolnicu S Hoang L Soslow RA. Recent advances in invasive adenocarcinoma of the cervix. Virchows Archiv: an Int J Pathol. (2019) 475:537–49. doi: 10.1007/s00428-019-02601-0, PMID: 31209635 PMC6864265

[111] Kim SH Basili T Dopeso H Da Cruz Paula A Bi R Issa Bhaloo S. Recurrent wwtr1 S89w mutations and hippo pathway deregulation in clear cell carcinomas of the cervix. J Pathol. (2022) 257:635–49. doi: 10.1002/path.5910, PMID: 35411948 PMC9881397

[112] Ortiz MA Mikhailova T Li X Porter BA Bah A Kotula L. Src family kinases, adaptor proteins and the actin cytoskeleton in epithelial-to-mesenchymal transition. Cell communication signaling: CCS. (2021) 19:67. doi: 10.1186/s12964-021-00750-x, PMID: 34193161 PMC8247114

[113] Liu Y Ren M Tan X Hu L. Distinct changes in the expression taz are associated with normal cervix and human cervical cancer. J Cancer. (2018) 9:4263–70. doi: 10.7150/jca.26623, PMID: 30519328 PMC6277613

[114] Li S Sampson C Liu C Piao HL Liu HX. Integrin signaling in cancer: bidirectional mechanisms and therapeutic opportunities. Cell communication signaling: CCS. (2023) 21:266. doi: 10.1186/s12964-023-01264-4, PMID: 37770930 PMC10537162

[115] Li Q Lan T Xie J Lu Y Zheng D Su B. Integrin-mediated tumorigenesis and its therapeutic applications. Front Oncol. (2022) 12:812480. doi: 10.3389/fonc.2022.812480, PMID: 35223494 PMC8873568

[116] Zhan P Liu L Liu B Mao XG. Expression of integrin B1 and its significance in squamous cell carcinoma of the cervix. Mol Med Rep. (2014) 9:2473–8. doi: 10.3892/mmr.2014.2134, PMID: 24718718

[117] Jiang X Maruyama J Iwasa H Arimoto-Matsuzaki K Nishina H Hata Y. Heat shock induces the nuclear accumulation of yap1 via src. Exp Cell Res. (2021) 399:112439. doi: 10.1016/j.yexcr.2020.112439, PMID: 33359469

[118] Zhu H Zhu H Tian M Wang D He J Xu T. DNA methylation and hydroxymethylation in cervical cancer: diagnosis, prognosis and treatment. Front Genet. (2020) 11:347. doi: 10.3389/fgene.2020.00347, PMID: 32328088 PMC7160865

[119] Skvortsova K Stirzaker C Taberlay P. The DNA methylation landscape in cancer. Essays Biochem. (2019) 63:797–811. doi: 10.1042/ebc20190037, PMID: 31845735 PMC6923322

[120] Han Y Ji L Guan Y Ma M Li P Xue Y. An epigenomic landscape of cervical intraepithelial neoplasia and cervical cancer using single-base resolution methylome and hydroxymethylome. Clin Trans Med. (2021) 11:e498. doi: 10.1002/ctm2.498, PMID: 34323415 PMC8288011

[121] Yang S Wu Y Wang S Xu P Deng Y Wang M. Hpv-related methylation-based reclassification and risk stratification of cervical cancer. Mol Oncol. (2020) 14:2124–41. doi: 10.1002/1878-0261.12709, PMID: 32408396 PMC7463306

[122] Pu M Xiao X Lv S Ran D Huang Q Zhou M. Mettl3-dependent dlg2 inhibits the Malignant progression of cervical cancer by inactivating the hippo/yap signaling. Hereditas. (2025) 162:9. doi: 10.1186/s41065-025-00365-z, PMID: 39856747 PMC11762078

[123] Saito Y Desai RR Muthuswamy SK. Reinterpreting polarity and cancer: the changing landscape from tumor suppression to tumor promotion. Biochim Biophys Acta Rev Cancer. (2018) 1869:103–16. doi: 10.1016/j.bbcan.2017.12.001, PMID: 29369778

[124] Li S Yan G Liu W Li C Wang X. Circ0106714 inhibits tumorigenesis of colorectal cancer by sponging mir-942-5p and releasing dlg2 via hippo-yap signaling. Mol carcinogenesis. (2020) 59:1323–42. doi: 10.1002/mc.23259, PMID: 33128289

[125] Griffin N Marsland M Roselli S Oldmeadow C Attia J Walker MM. The receptor tyrosine kinase trka is increased and targetable in her2-positive breast cancer. Biomolecules. (2020) 10:1329. doi: 10.3390/biom10091329, PMID: 32957504 PMC7564775

[126] Kobayashi K Ando M Saito Y Kondo K Omura G Shinozaki-Ushiku A. Nerve growth factor signals as possible pathogenic biomarkers for perineural invasion in adenoid cystic carcinoma. Otolaryngology–head Neck surgery: Off J Am Acad Otolaryngology-Head Neck Surg. (2015) 153:218–24. doi: 10.1177/0194599815584762, PMID: 25968060

[127] Yu EH Lui MT Tu HF Wu CH Lo WL Yang CC. Oral carcinoma with perineural invasion has higher nerve growth factor expression and worse prognosis. Oral Dis. (2014) 20:268–74. doi: 10.1111/odi.12101, PMID: 23556997

[128] Wei YS Yao DS Long Y. Evaluation of the association between perineural invasion and clinical and histopathological features of cervical cancer. Mol Clin Oncol. (2016) 5:307–11. doi: 10.3892/mco.2016.941, PMID: 27588197 PMC4998037

[129] Cui L Shi Y Zhang GN. Perineural invasion as a prognostic factor for cervical cancer: A systematic review and meta-analysis. Arch gynecology obstetrics. (2015) 292:13–9. doi: 10.1007/s00404-015-3627-z, PMID: 25637504

[130] Faulkner S Griffin N Rowe CW Jobling P Lombard JM Oliveira SM. Nerve growth factor and its receptor tyrosine kinase trka are overexpressed in cervical squamous cell carcinoma. FASEB bioAdvances. (2020) 2:398–408. doi: 10.1096/fba.2020-00016, PMID: 32676580 PMC7354692

[131] Long Y Yao DS Wei YS Wu GT. Effects of nerve growth factor expression on perineural invasion and worse prognosis in early-stage cervical cancer. Chin Med J. (2018) 131:2360–3. doi: 10.4103/0366-6999.241808, PMID: 30246726 PMC6166468

[132] Elsahwi KS Barber E Illuzzi J Buza N Ratner E Silasi DA. The significance of perineural invasion in early-stage cervical cancer. Gynecologic Oncol. (2011) 123:561–4. doi: 10.1016/j.ygyno.2011.08.028, PMID: 21968340

[133] Wang L Li J Wang R Chen H Wang R Wang W. Ngf signaling interacts with the hippo/yap pathway to regulate cervical cancer progression. Front Oncol. (2021) 11:688794. doi: 10.3389/fonc.2021.688794, PMID: 34722240 PMC8552705

[134] Oo WM Hunter DJ. Nerve growth factor (Ngf) inhibitors and related agents for chronic musculoskeletal pain: A comprehensive review. BioDrugs: Clin immunotherapeutics biopharmaceuticals Gene Ther. (2021) 35:611–41. doi: 10.1007/s40259-021-00504-8, PMID: 34807432

[135] Fry AM Bayliss R Roig J. Mitotic regulation by nek kinase networks. Front Cell Dev Biol. (2017) 5:102. doi: 10.3389/fcell.2017.00102, PMID: 29250521 PMC5716973

[136] Fang Y Kong Y Xi J Zhu M Zhu T Jiang T. Preclinical activity of mbm-5 in gastrointestinal cancer by inhibiting nek2 kinase activity. Oncotarget. (2016) 7:79327–41. doi: 10.18632/oncotarget.12687, PMID: 27764815 PMC5346717

[137] Li G Zhong Y Shen Q Zhou Y Deng X Li C. Nek2 serves as a prognostic biomarker for hepatocellular carcinoma. Int J Oncol. (2017) 50:405–13. doi: 10.3892/ijo.2017.3837, PMID: 28101574 PMC5238800

[138] Panchal NK Evan Prince S. The nek family of serine/threonine kinases as a biomarker for cancer. Clin Exp Med. (2023) 23:17–30. doi: 10.1007/s10238-021-00782-0, PMID: 35037094

[139] Hayward DG Clarke RB Faragher AJ Pillai MR Hagan IM Fry AM. The centrosomal kinase nek2 displays elevated levels of protein expression in human breast cancer. Cancer Res. (2004) 64:7370–6. doi: 10.1158/0008-5472.can-04-0960, PMID: 15492258

[140] Xu T Zeng Y Shi L Yang Q Chen Y Wu G. Targeting nek2 impairs oncogenesis and radioresistance via inhibiting the wnt1/B-catenin signaling pathway in cervical cancer. J Exp Clin Cancer research: CR. (2020) 39:183. doi: 10.1186/s13046-020-01659-y, PMID: 32907622 PMC7488040

[141] Xue JM Liu Y Wan LH Zhu YX. Comprehensive analysis of differential gene expression to identify common gene signatures in multiple cancers. Med Sci monitor: Int Med J Exp Clin Res. (2020) 26:e919953. doi: 10.12659/msm.919953, PMID: 32035007 PMC7027371

[142] van Dam PA Rolfo C Ruiz R Pauwels P Van Berckelaer C Trinh XB. Potential new biomarkers for squamous carcinoma of the uterine cervix. ESMO Open. (2018) 3:e000352. doi: 10.1136/esmoopen-2018-000352, PMID: 30018810 PMC6045706

[143] Zhang YR Zheng PS. Nek2 inactivates the hippo pathway to advance the proliferation of cervical cancer cells by cooperating with stripak complexes. Cancer Lett. (2022) 549:215917. doi: 10.1016/j.canlet.2022.215917, PMID: 36115593

[144] Rankin EB Nam JM Giaccia AJ. Hypoxia: signaling the metastatic cascade. Trends Cancer. (2016) 2:295–304. doi: 10.1016/j.trecan.2016.05.006, PMID: 28741527 PMC5808868

[145] Matsuo Y Ding Q Desaki R Maemura K Mataki Y Shinchi H. Hypoxia inducible factor-1 alpha plays a pivotal role in hepatic metastasis of pancreatic cancer: an immunohistochemical study. J hepato-biliary-pancreatic Sci. (2014) 21:105–12. doi: 10.1002/jhbp.6, PMID: 23798470

[146] Mokoala KMG Lawal IO Maserumule LC Bida M Maes A Ndlovu H. Correlation between [(68)Ga]Ga-fapi-46 pet imaging and hif-1α Immunohistochemical analysis in cervical cancer: proof-of-concept. Cancers. (2023) 15:3953. doi: 10.3390/cancers15153953, PMID: 37568769 PMC10417683

[147] Yin CL Ma YJ. The regulatory mechanism of hypoxia-inducible factor 1 and its clinical significance. Curr Mol Pharmacol. (2024) 17:e18761429266116. doi: 10.2174/0118761429266116231123160809, PMID: 38389420

[148] Infantino V Santarsiero A Convertini P Todisco S Iacobazzi V. Cancer cell metabolism in hypoxia: role of hif-1 as key regulator and therapeutic target. Int J Mol Sci. (2021) 22:5703. doi: 10.3390/ijms22115703, PMID: 34071836 PMC8199012

[149] Qannita RA Alalami AI Harb AA Aleidi SM Taneera J Abu-Gharbieh E. Targeting hypoxia-inducible factor-1 (Hif-1) in cancer: emerging therapeutic strategies and pathway regulation. Pharm (Basel Switzerland). (2024) 17:195. doi: 10.3390/ph17020195, PMID: 38399410 PMC10892333

[150] Yu T Tang B Sun X. Development of inhibitors targeting hypoxia-inducible factor 1 and 2 for cancer therapy. Yonsei Med J. (2017) 58:489–96. doi: 10.3349/ymj.2017.58.3.489, PMID: 28332352 PMC5368132

[151] Méndez-Blanco C Fernández-Palanca P Fondevila F González-Gallego J Mauriz JL. Prognostic and clinicopathological significance of hypoxia-inducible factors 1α and 2α in hepatocellular carcinoma: A systematic review with meta-analysis. Ther Adv Med Oncol. (2021) 13:1758835920987071. doi: 10.1177/1758835920987071, PMID: 33613697 PMC7874357

[152] Rodríguez ME Catrinacio C Ropolo A Rivarola VA Vaccaro MI. A novel hif-1α/vmp1-autophagic pathway induces resistance to photodynamic therapy in colon cancer cells. Photochemical photobiological sciences: Off J Eur Photochem Assoc Eur Soc Photobiol. (2017) 16:1631–42. doi: 10.1039/c7pp00161d, PMID: 28936522

[153] Abudoukerimu A Hasimu A Abudoukerimu A Tuerxuntuoheti G Huang Y Wei J. Hif-1α Regulates the progression of cervical cancer by targeting yap/taz. J Oncol. (2022) 2022:3814809. doi: 10.1155/2022/3814809, PMID: 35664561 PMC9159877

[154] Donini C D’Ambrosio L Grignani G Aglietta M Sangiolo D. Next generation immune-checkpoints for cancer therapy. J Thorac Dis. (2018) 10:S1581–s601. doi: 10.21037/jtd.2018.02.79, PMID: 29951308 PMC5994499

[155] Janse van Rensburg HJ Azad T Ling M Hao Y Snetsinger B Khanal P. The hippo pathway component taz promotes immune evasion in human cancer through pd-L1. Cancer Res. (2018) 78:1457–70. doi: 10.1158/0008-5472.can-17-3139, PMID: 29339539

[156] Feng J Yang H Zhang Y Wei H Zhu Z Zhu B. Tumor cell-derived lactate induces taz-dependent upregulation of pd-L1 through gpr81 in human lung cancer cells. Oncogene. (2017) 36:5829–39. doi: 10.1038/onc.2017.188, PMID: 28604752

[157] Nasser MW Wani NA Ahirwar DK Powell CA Ravi J Elbaz M. Rage mediates S100a7-induced breast cancer growth and metastasis by modulating the tumor microenvironment. Cancer Res. (2015) 75:974–85. doi: 10.1158/0008-5472.can-14-2161, PMID: 25572331 PMC4359968

[158] Fan JJ Hsu WH Lee KH Chen KC Lin CW Lee YA. Dietary flavonoids luteolin and quercetin inhibit migration and invasion of squamous carcinoma through reduction of src/stat3/S100a7 signaling. Antioxidants (Basel Switzerland). (2019) 8:557. doi: 10.3390/antiox8110557, PMID: 31731716 PMC6912538

[159] Sood A Mishra D Kharbanda OP Chauhan SS Gupta SD Deo SSV. Role of S100 A7 as a diagnostic biomarker in oral potentially Malignant disorders and oral cancer. J Oral Maxillofac pathology: JOMFP. (2022) 26:166–72. doi: 10.4103/jomfp.jomfp_402_20, PMID: 35968185 PMC9364636

[160] Liu Y Bunston C Hodson N Resaul J Sun PH Cai S. Psoriasin promotes invasion, aggregation and survival of pancreatic cancer cells; association with disease progression. Int J Oncol. (2017) 50:1491–500. doi: 10.3892/ijo.2017.3953, PMID: 28393239 PMC5403466

[161] Tian T Li X Hua Z Ma J Wu X Liu Z. S100a7 promotes the migration, invasion and metastasis of human cervical cancer cells through epithelial-mesenchymal transition. Oncotarget. (2017) 8:24964–77. doi: 10.18632/oncotarget.15329, PMID: 28212564 PMC5421902

[162] Youssef KK Nieto MA. Epithelial-mesenchymal transition in tissue repair and degeneration. Nat Rev Mol Cell Biol. (2024) 25:720–39. doi: 10.1038/s41580-024-00733-z, PMID: 38684869

[163] Kong F Li Y Hu E Wang R Wang J Liu J. The characteristic of S100a7 induction by the hippo-yap pathway in cervical and glossopharyngeal squamous cell carcinoma. PloS One. (2016) 11:e0167080. doi: 10.1371/journal.pone.0167080, PMID: 27907036 PMC5132200

[164] Akimoto T Hunter NR Buchmiller L Mason K Ang KK Milas L. Inverse relationship between epidermal growth factor receptor expression and radiocurability of murine carcinomas. Clin Cancer research: an Off J Am Assoc Cancer Res. (1999) 5:2884–90., PMID: 10537357

[165] Huang SM Harari PM. Modulation of radiation response after epidermal growth factor receptor blockade in squamous cell carcinomas: inhibition of damage repair, cell cycle kinetics, and tumor angiogenesis. Clin Cancer research: an Off J Am Assoc Cancer Res. (2000) 6:2166–74., PMID: 10873065

[166] Milas L Mason K Hunter N Petersen S Yamakawa M Ang K. *In vivo* enhancement of tumor radioresponse by C225 antiepidermal growth factor receptor antibody. Clin Cancer research: an Off J Am Assoc Cancer Res. (2000) 6:701–8., PMID: 10690556

[167] Bianco C Bianco R Tortora G Damiano V Guerrieri P Montemaggi P. Antitumor activity of combined treatment of human cancer cells with ionizing radiation and anti-epidermal growth factor receptor monoclonal antibody C225 plus type I protein kinase a antisense oligonucleotide. Clin Cancer research: an Off J Am Assoc Cancer Res. (2000) 6:4343–50., PMID: 11106252

[168] Santin AD Sill MW McMeekin DS Leitao MM Jr. Brown J Sutton GP. Phase ii trial of cetuximab in the treatment of persistent or recurrent squamous or non-squamous cell carcinoma of the cervix: A gynecologic oncology group study. Gynecologic Oncol. (2011) 122:495–500. doi: 10.1016/j.ygyno.2011.05.040, PMID: 21684583 PMC3152667

[169] Farley J Sill MW Birrer M Walker J Schilder RJ Thigpen JT. Phase ii study of cisplatin plus cetuximab in advanced, recurrent, and previously treated cancers of the cervix and evaluation of epidermal growth factor receptor immunohistochemical expression: A gynecologic oncology group study. Gynecologic Oncol. (2011) 121:303–8. doi: 10.1016/j.ygyno.2011.01.030, PMID: 21329967 PMC3081894

[170] Nogueira-Rodrigues A Moralez G Grazziotin R Carmo CC Small IA Alves FV. Phase 2 trial of erlotinib combined with cisplatin and radiotherapy in patients with locally advanced cervical cancer. Cancer. (2014) 120:1187–93. doi: 10.1002/cncr.28471, PMID: 24615735

[171] Huang J Theelen W Belcaid Z Najjar M van der Geest D Singh D. Combination of pembrolizumab and radiotherapy induces systemic antitumor immune responses in immunologically cold non-small cell lung cancer. Nat Cancer. (2025). doi: 10.1038/s43018-025-01018-w, PMID: 40696153 PMC12559004

[172] Lorusso D Colombo N Dubot C Cáceres MV Hasegawa K Shapira-Frommer R. Pembrolizumab plus chemotherapy for advanced and recurrent cervical cancer: final analysis according to bevacizumab use in the randomized keynote-826 study. Ann Oncol Off J Eur Soc Med Oncol. (2025) 36:65–75. doi: 10.1016/j.annonc.2024.10.002, PMID: 39393777

[173] Lorusso D Xiang Y Hasegawa K Scambia G Leiva M Ramos-Elias P. Pembrolizumab or placebo with chemoradiotherapy followed by pembrolizumab or placebo for newly diagnosed, high-risk, locally advanced cervical cancer (Engot-cx11/gog-3047/keynote-A18): A randomised, double-blind, phase 3 clinical trial. Lancet (London England). (2024) 403:1341–50. doi: 10.1016/s0140-6736(24)00317-9, PMID: 38521086

[174] Lorusso D Xiang Y Hasegawa K Scambia G Leiva M Ramos-Elias P. Pembrolizumab or placebo with chemoradiotherapy followed by pembrolizumab or placebo for newly diagnosed, high-risk, locally advanced cervical cancer (Engot-cx11/gog-3047/keynote-A18): overall survival results from a randomised, double-blind, placebo-controlled, phase 3 trial. Lancet (London England). (2024) 404:1321–32. doi: 10.1016/s0140-6736(24)01808-7, PMID: 39288779

[175] Randall L Xiang Y Matsumoto T Giannarelli D Milla DP Lopez KA. Patient-reported outcomes from the phase 3, randomized, double-blind, placebo-controlled engot-cx11/gog-3047/keynote-A18 study of pembrolizumab plus concurrent chemoradiotherapy in participants with high-risk locally advanced cervical cancer. Gynecologic Oncol. (2025) 199:88–95. doi: 10.1016/j.ygyno.2025.06.003, PMID: 40592026

[176] Oaknin A Monk BJ de Melo AC Kim HS Kim YM Lisyanskaya AS. Cemiplimab in recurrent cervical cancer: final analysis of overall survival in the phase iii empower-cervical 1/gog-3016/engot-cx9 trial. Eur J Cancer (Oxford England: 1990). (2025) 216:115146. doi: 10.1016/j.ejca.2024.115146, PMID: 39798514

[177] Oaknin A Monk BJ Vergote I Cristina de Melo A Kim YM Lisyanskaya AS. Empower cervical-1: effects of cemiplimab versus chemotherapy on patient-reported quality of life, functioning and symptoms among women with recurrent cervical cancer. Eur J Cancer (Oxford England: 1990). (2022) 174:299–309. doi: 10.1016/j.ejca.2022.03.016, PMID: 35922251

[178] An J Tang J Li BX Xiong H Qiu H Luo L. Efficacy and safety of the anti-pd-L1 mab socazolimab for recurrent or metastatic cervical cancer: A phase I dose-escalation and expansion study. Clin Cancer research: an Off J Am Assoc Cancer Res. (2022) 28:5098–106. doi: 10.1158/1078-0432.ccr-22-1280, PMID: 36136294

[179] Juric D Rodon J Tabernero J Janku F Burris HA Schellens JHM. Phosphatidylinositol 3-kinase A-selective inhibition with alpelisib (Byl719) in pik3ca-altered solid tumors: results from the first-in-human study. J Clin Oncol Off J Am Soc Clin Oncol. (2018) 36:1291–9. doi: 10.1200/jco.2017.72.7107, PMID: 29401002 PMC5920739

[180] Djebali S Davis CA Merkel A Dobin A Lassmann T Mortazavi A. Landscape of transcription in human cells. Nature. (2012) 489:101–8. doi: 10.1038/nature11233, PMID: 22955620 PMC3684276

[181] Jiang X Ma N Wang D Li F He R Li D. Metformin inhibits tumor growth by regulating multiple mirnas in human cholangiocarcinoma. Oncotarget. (2015) 6:3178–94. doi: 10.18632/oncotarget.3063, PMID: 25605008 PMC4413646

[182] Vance KW Sansom SN Lee S Chalei V Kong L Cooper SE. The long non-coding rna paupar regulates the expression of both local and distal genes. EMBO J. (2014) 33:296–311. doi: 10.1002/embj.201386225, PMID: 24488179 PMC3983687

[183] Huang B Song JH Cheng Y Abraham JM Ibrahim S Sun Z. Long non-coding antisense rna krt7-as is activated in gastric cancers and supports cancer cell progression by increasing krt7 expression. Oncogene. (2016) 35:4927–36. doi: 10.1038/onc.2016.25, PMID: 26876208 PMC4985510

[184] Mattick JS Amaral PP Carninci P Carpenter S Chang HY Chen LL. Long non-coding rnas: definitions, functions, challenges and recommendations. Nat Rev Mol Cell Biol. (2023) 24:430–47. doi: 10.1038/s41580-022-00566-8, PMID: 36596869 PMC10213152

[185] Han X Li B Zhang S. Mir503hg: A potential diagnostic and therapeutic target in human diseases. Biomedicine pharmacotherapy = Biomedecine pharmacotherapie. (2023) 160:114314. doi: 10.1016/j.biopha.2023.114314, PMID: 36736276

[186] Rysz J Konecki T Franczyk B Ławiński J Gluba-Brzózka A. The role of long noncoding rna (Lncrnas) biomarkers in renal cell carcinoma. Int J Mol Sci. (2022) 24:643. doi: 10.3390/ijms24010643, PMID: 36614082 PMC9820502

[187] Al-Shehri A Bakhashab S. Oncogenic long noncoding rnas in prostate cancer, osteosarcoma, and metastasis. Biomedicines. (2023) 11:633. doi: 10.3390/biomedicines11020633, PMID: 36831169 PMC9953056

[188] Kumar A Girisa S Alqahtani MS Abbas M Hegde M Sethi G. Targeting autophagy using long non-coding rnas (Lncrnas): new landscapes in the arena of cancer therapeutics. Cells. (2023) 12:810. doi: 10.3390/cells12050810, PMID: 36899946 PMC10000689

[189] O’Brien J Hayder H Zayed Y Peng C. Overview of microrna biogenesis, mechanisms of actions, and circulation. Front Endocrinol. (2018) 9:402. doi: 10.3389/fendo.2018.00402, PMID: 30123182 PMC6085463

[190] Wang J Mei J Ren G. Plant micrornas: biogenesis, homeostasis, and degradation. Front Plant Sci. (2019) 10:360. doi: 10.3389/fpls.2019.00360, PMID: 30972093 PMC6445950

[191] Lin S Gregory RI. Microrna biogenesis pathways in cancer. Nat Rev Cancer. (2015) 15:321–33. doi: 10.1038/nrc3932, PMID: 25998712 PMC4859809

[192] Kapplingattu SV Bhattacharya S Adlakha YK. Mirnas as major players in brain health and disease: current knowledge and future perspectives. Cell Death Discov. (2025) 11:7. doi: 10.1038/s41420-024-02283-x, PMID: 39805813 PMC11729916

[193] Zhang Z Zhou X Li J Meng Q Zheng P. Lncrna hotair promotes the migration and invasion of cervical cancer through dnmt3b/lats1/yap1 ps127 axis. Reprod Biol. (2024) 24:100893. doi: 10.1016/j.repbio.2024.100893, PMID: 38754347

[194] Zhang J Zhang P Wang L Piao HL Ma L. Long non-coding rna hotair in carcinogenesis and metastasis. Acta Biochim Biophys Sin. (2014) 46:1–5. doi: 10.1093/abbs/gmt117, PMID: 24165275 PMC3869294

[195] Raju GSR Pavitra E Bandaru SS Varaprasad GL Nagaraju GP Malla RR. Hotair: A potential metastatic, drug-resistant and prognostic regulator of breast cancer. Mol Cancer. (2023) 22:65. doi: 10.1186/s12943-023-01765-3, PMID: 36997931 PMC10061914

[196] Xin X Li Q Fang J Zhao T. Lncrna hotair: A potential prognostic factor and therapeutic target in human cancers. Front Oncol. (2021) 11:679244. doi: 10.3389/fonc.2021.679244, PMID: 34367966 PMC8340021

[197] Li P Wang J Zhi L Cai F. Linc00887 suppresses tumorigenesis of cervical cancer through regulating the mir-454-3p/frmd6-hippo axis. Cancer Cell Int. (2021) 21:33. doi: 10.1186/s12935-020-01730-w, PMID: 33413358 PMC7792119

[198] Morgan EL Patterson MR Ryder EL Lee SY Wasson CW Harper KL. Microrna-18a targeting of the stk4/mst1 tumour suppressor is necessary for transformation in hpv positive cervical cancer. PloS Pathog. (2020) 16:e1008624. doi: 10.1371/journal.ppat.1008624, PMID: 32555725 PMC7326282

[199] Nazimek K Bryniarski K. Perspectives in manipulating evs for therapeutic applications: focus on cancer treatment. Int J Mol Sci. (2020) 21:4623. doi: 10.3390/ijms21134623, PMID: 32610582 PMC7369858

[200] Wang W Wu L Tian J Yan W Qi C Liu W. Cervical cancer cells-derived extracellular vesicles containing microrna-146a-5p affect actin dynamics to promote cervical cancer metastasis by activating the hippo-yap signaling pathway via wwc2. J Oncol. (2022) 2022:4499876. doi: 10.1155/2022/4499876, PMID: 35799607 PMC9256433

[201] Di Fiore R Suleiman S Drago-Ferrante R Subbannayya Y Pentimalli F Giordano A. Cancer stem cells and their possible implications in cervical cancer: A short review. Int J Mol Sci. (2022) 23:5167. doi: 10.3390/ijms23095167, PMID: 35563557 PMC9106065

[202] Bi L Ma F Tian R Zhou Y Lan W Song Q. Ajuba increases the cisplatin resistance through hippo pathway in cervical cancer. Gene. (2018) 644:148–54. doi: 10.1016/j.gene.2017.11.017, PMID: 29126926

[203] Das Thakur M Feng Y Jagannathan R Seppa MJ Skeath JB Longmore GD. Ajuba lim proteins are negative regulators of the hippo signaling pathway. Curr biology: CB. (2010) 20:657–62. doi: 10.1016/j.cub.2010.02.035, PMID: 20303269 PMC2855397

[204] Wang H Yang Y Zhang E Wang D Cai W Li C. Lncrna pgm5-as1 impairs the resistance of cervical cancer to cisplatin by regulating the hippo and pi3k-akt pathways. Biochem Genet. (2024). doi: 10.1007/s10528-024-11011-0, PMID: 39733221

[205] Liang M Sheng L Ke Y Wu Z. The research progress on radiation resistance of cervical cancer. Front Oncol. (2024) 14:1380448. doi: 10.3389/fonc.2024.1380448, PMID: 38651153 PMC11033433

[206] Moreno-Acosta P Vallard A Carrillo S Gamboa O Romero-Rojas A Molano M. Biomarkers of resistance to radiation therapy: A prospective study in cervical carcinoma. Radiat Oncol (London England). (2017) 12:120. doi: 10.1186/s13014-017-0856-2, PMID: 28716107 PMC5514482

[207] Shi ZY Li CY Chen RY Shi JJ Liu YJ Lu JF. The emerging role of deubiquitylating enzyme usp21 as a potential therapeutic target in cancer. Bioorganic Chem. (2024) 147:107400. doi: 10.1016/j.bioorg.2024.107400, PMID: 38688196

[208] Kaushal K Antao AM Kim KS Ramakrishna S. Deubiquitinating enzymes in cancer stem cells: functions and targeted inhibition for cancer therapy. Drug Discov Today. (2018) 23:1974–82. doi: 10.1016/j.drudis.2018.05.035, PMID: 29864528

[209] Li Z Liu X Yu H Wang S Zhao S Jiang G. Usp21 regulates hippo signaling to promote radioresistance by deubiquitinating foxm1 in cervical cancer. Hum Cell. (2022) 35:333–47. doi: 10.1007/s13577-021-00650-9, PMID: 34825342

[210] Eisinger-Mathason TS Mucaj V Biju KM Nakazawa MS Gohil M Cash TP. Deregulation of the hippo pathway in soft-tissue sarcoma promotes foxm1 expression and tumorigenesis. Proc Natl Acad Sci United States America. (2015) 112:E3402–11. doi: 10.1073/pnas.1420005112, PMID: 26080399 PMC4491775

[211] Li X Lin YY Tan JY Liu KL Shen XL Hu YJ. an anticancer agent, inhibits yap via transcriptional and post-translational regulation. Pharm Biol. (2021) 59:619–28. doi: 10.1080/13880209.2021.1923759, PMID: 34010589 PMC8143639

[212] Wang C Zhu X Feng W Yu Y Jeong K Guo W. Verteporfin Inhibits Yap Function through up-Regulating 14-3-3σ Sequestering Yap in the Cytoplasm. Am J Cancer Res. (2016) 6:27–37., PMID: 27073720 PMC4759394

[213] Boda D Docea AO Calina D Ilie MA Caruntu C Zurac S. Human papilloma virus: apprehending the link with carcinogenesis and unveiling new research avenues (Review). Int J Oncol. (2018) 52:637–55. doi: 10.3892/ijo.2018.4256, PMID: 29393378 PMC5807043

[214] Ryndock EJ Meyers C. A risk for non-sexual transmission of human papillomavirus? Expert Rev anti-infective Ther. (2014) 12:1165–70. doi: 10.1586/14787210.2014.959497, PMID: 25199987

[215] Bhattacharjee R Das SS Biswal SS Nath A Das D Basu A. Mechanistic role of hpv-associated early proteins in cervical cancer: molecular pathways and targeted therapeutic strategies. Crit Rev oncology/hematology. (2022) 174:103675. doi: 10.1016/j.critrevonc.2022.103675, PMID: 35381343

[216] Wang D He J Dong J Meyer TF Xu T. The hippo pathway in gynecological Malignancies. Am J Cancer Res. (2020) 10:610–29. PMC7061741 32195031

[217] Patterson MR Cogan JA Cassidy R Theobald DA Wang M Scarth JA. The hippo pathway transcription factors yap and taz play hpv-type dependent roles in cervical cancer. Nat Commun. (2024) 15:5809. doi: 10.1038/s41467-024-49965-9, PMID: 38987584 PMC11237029

[218] Maehama T Nishio M Otani J Mak TW Suzuki A. The role of hippo-yap signaling in squamous cell carcinomas. Cancer Sci. (2021) 112:51–60. doi: 10.1111/cas.14725, PMID: 33159406 PMC7780025

[219] Cohen PA Jhingran A Oaknin A Denny L. Cervical cancer. Lancet (London England). (2019) 393:169–82. doi: 10.1016/s0140-6736(18)32470-x, PMID: 30638582

[220] Hill RJ Bona N Smink J Webb HK Crisp A Garaycoechea JI. P53 regulates diverse tissue-specific outcomes to endogenous DNA damage in mice. Nat Commun. (2024) 15:2518. doi: 10.1038/s41467-024-46844-1, PMID: 38514641 PMC10957910

[221] Kashyap VK Dan N Chauhan N Wang Q Setua S Nagesh PKB. Veru-111 suppresses tumor growth and metastatic phenotypes of cervical cancer cells through the activation of P53 signaling pathway. Cancer Lett. (2020) 470:64–74. doi: 10.1016/j.canlet.2019.11.035, PMID: 31809801 PMC8059100

[222] Hietanen S Lain S Krausz E Blattner C Lane DP. Activation of P53 in cervical carcinoma cells by small molecules. Proc Natl Acad Sci United States America. (2000) 97:8501–6. doi: 10.1073/pnas.97.15.8501, PMID: 10900010 PMC26977

[223] Bai N Zhang C Liang N Zhang Z Chang A Yin J. Yes-associated protein (Yap) increases chemosensitivity of hepatocellular carcinoma cells by modulation of P53. Cancer Biol Ther. (2013) 14:511–20. doi: 10.4161/cbt.24345, PMID: 23760493 PMC3813567

[224] Furth N Aylon Y. The lats1 and lats2 tumor suppressors: beyond the hippo pathway. Cell Death differentiation. (2017) 24:1488–501. doi: 10.1038/cdd.2017.99, PMID: 28644436 PMC5563998

[225] Aylon Y Oren M. The paradox of P53: what, how, and why? Cold Spring Harbor Perspect Med. (2016) 6:a026328. doi: 10.1101/cshperspect.a026328, PMID: 27413116 PMC5046691

[226] Aylon Y Michael D Shmueli A Yabuta N Nojima H Oren M. A positive feedback loop between the P53 and lats2 tumor suppressors prevents tetraploidization. Genes Dev. (2006) 20:2687–700. doi: 10.1101/gad.1447006, PMID: 17015431 PMC1578695

[227] Nováková J Talacko P Novák P Vališ K. The mek-erk-mst1 axis potentiates the activation of the extrinsic apoptotic pathway during gdc-0941 treatment in jurkat T cells. Cells. (2019) 8:191. doi: 10.3390/cells8020191, PMID: 30795621 PMC6406719

[228] Lee J Lee N Han HD Lee Y. Hypoxic induction of apoptosis occurs through hif-1α and accompanies mammalian sterile 20-like kinase 2 cleavage in human endometrial adenocarcinoma ishikawa cells. Biochem Biophys Res Commun. (2022) 604:104–8. doi: 10.1016/j.bbrc.2022.03.016, PMID: 35303675

[229] Sharma A Kansara S Mahajan M Yadav B Garg M Pandey AK. Long non-coding rnas orchestrate various molecular and cellular processes by modulating epithelial-mesenchymal transition in head and neck squamous cell carcinoma. Biochim Biophys Acta Mol basis Dis. (2021) 1867:166240. doi: 10.1016/j.bbadis.2021.166240, PMID: 34363933

[230] Xiao Y Yu D. Tumor microenvironment as a therapeutic target in cancer. Pharmacol Ther. (2021) 221:107753. doi: 10.1016/j.pharmthera.2020.107753, PMID: 33259885 PMC8084948

[231] Bergman E Goldbart R Traitel T Amar-Lewis E Zorea J Yegodayev K. Cell stiffness predicts cancer cell sensitivity to ultrasound as a selective superficial cancer therapy. Bioengineering Trans Med. (2021) 6:e10226. doi: 10.1002/btm2.10226, PMID: 34589601 PMC8459597

[232] Lampi MC Reinhart-King CA. Targeting extracellular matrix stiffness to attenuate disease: from molecular mechanisms to clinical trials. Sci Trans Med. (2018) 10:eaao0475. doi: 10.1126/scitranslmed.aao0475, PMID: 29298864

[233] Yang L Li J Zang G Song S Sun Z Li X. Pin1/yap pathway mediates matrix stiffness-induced epithelial-mesenchymal transition driving cervical cancer metastasis via a non-hippo mechanism. Bioengineering Trans Med. (2023) 8:e10375. doi: 10.1002/btm2.10375, PMID: 36684109 PMC9842039

[234] Kim J Kim YH Kim J Park DY Bae H Lee DH. Yap/taz regulates sprouting angiogenesis and vascular barrier maturation. J Clin Invest. (2017) 127:3441–61. doi: 10.1172/jci93825, PMID: 28805663 PMC5669570

[235] Wang X Freire Valls A Schermann G Shen Y Moya IM Castro L. Yap/taz orchestrate vegf signaling during developmental angiogenesis. Dev Cell. (2017) 42:462–78.e7. doi: 10.1016/j.devcel.2017.08.002, PMID: 28867486

[236] Grivennikov SI Greten FR Karin M. Immunity, inflammation, and cancer. Cell. (2010) 140:883–99. doi: 10.1016/j.cell.2010.01.025, PMID: 20303878 PMC2866629

[237] Guo X Zhao Y Yan H Yang Y Shen S Dai X. Single tumor-initiating cells evade immune clearance by recruiting type ii macrophages. Genes Dev. (2017) 31:247–59. doi: 10.1101/gad.294348.116, PMID: 28223311 PMC5358722

[238] Huang YJ Yang CK Wei PL Huynh TT Whang-Peng J Meng TC. Ovatodiolide suppresses colon tumorigenesis and prevents polarization of M2 tumor-associated macrophages through yap oncogenic pathways. J Hematol Oncol. (2017) 10:60. doi: 10.1186/s13045-017-0421-3, PMID: 28241877 PMC5329923

[239] Pan Z Tian Y Cao C Niu G. The emerging role of yap/taz in tumor immunity. Mol Cancer research: MCR. (2019) 17:1777–86. doi: 10.1158/1541-7786.mcr-19-0375, PMID: 31308148

[240] Wang G Lu X Dey P Deng P Wu CC Jiang S. Targeting yap-dependent mdsc infiltration impairs tumor progression. Cancer Discov. (2016) 6:80–95. doi: 10.1158/2159-8290.cd-15-0224, PMID: 26701088 PMC4707102

[241] Stampouloglou E Cheng N Federico A Slaby E Monti S Szeto GL. Yap suppresses T-cell function and infiltration in the tumor microenvironment. PloS Biol. (2020) 18:e3000591. doi: 10.1371/journal.pbio.3000591, PMID: 31929526 PMC6980695

[242] Zhang JJ Zhang QS Li ZQ Zhou JW Du J. Metformin attenuates pd-L1 expression through activating hippo signaling pathway in colorectal cancer cells. Am J Trans Res. (2019) 11:6965–76., PMID: 31814900 PMC6895520

[243] Eksteen C Riedemann J Rass AM Plessis MD Botha MH van der Merwe FH. A review: genetic mutations as a key to unlocking drug resistance in cervical cancer. Cancer control: J Moffitt Cancer Center. (2024) 31:10732748241261539. doi: 10.1177/10732748241261539, PMID: 38881031 PMC11181891

[244] Galicia-Carmona T Arango-Bravo EA Coronel-Martínez JA Cetina-Pérez L Vanoye-Carlo EG Villalobos-Valencia R. Advanced, recurrent, and persistent cervical cancer management: in the era of immunotherapy. Front Oncol. (2024) 14:1392639. doi: 10.3389/fonc.2024.1392639, PMID: 39161386 PMC11330775

[245] Dogruluk T Tsang YH Espitia M Chen F Chen T Chong Z. Identification of variant-specific functions of pik3ca by rapid phenotyping of rare mutations. Cancer Res. (2015) 75:5341–54. doi: 10.1158/0008-5472.can-15-1654, PMID: 26627007 PMC4681596

[246] Wei Y Lin S Zhi W Chu T Liu B Peng T. Genomic analysis of cervical carcinoma identifies alpelisib as a therapeutic option for pik3ca-mutant cervical carcinoma via the pi3k/akt pathway. J Med Virol. (2023) 95:e28656. doi: 10.1002/jmv.28656, PMID: 36905114

[247] Berthold R Isfort I Erkut C Heinst L Grünewald I Wardelmann E. Fusion protein-driven igf-ir/pi3k/akt signals deregulate hippo pathway promoting oncogenic cooperation of yap1 and fus-ddit3 in myxoid liposarcoma. Oncogenesis. (2022) 11:20. doi: 10.1038/s41389-022-00394-7, PMID: 35459264 PMC9033823

[248] Harvey KF Tang TT. Targeting the hippo pathway in cancer. Nat Rev Drug Discov. (2025). doi: 10.1038/s41573-025-01234-0, PMID: 40588515

