# Supplementary figures and images for: Hippo signaling pathway in cervical cancer: insights into mechanisms and therapeutic potential
Source: Front Oncol. 2025 Nov 3;15:1662499. doi: 10.3389/fonc.2025.1662499 (PMC12620204; doi:10.3389/fonc.2025.1662499)

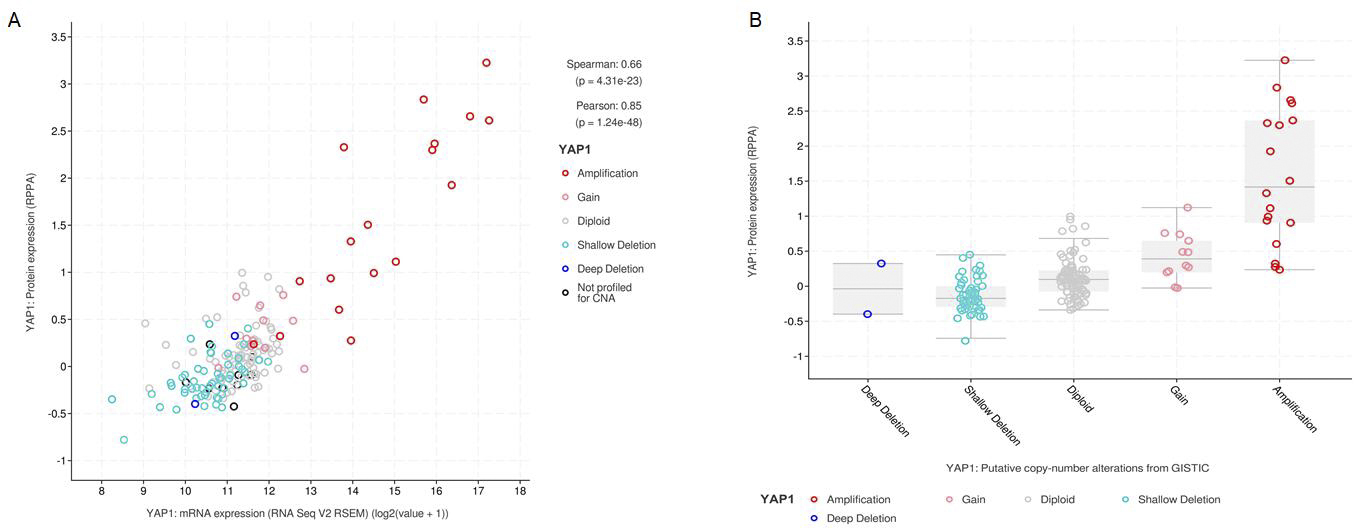

Supplement: Supplementary Figure 1 — YAP1 gene amplification drives its overexpression at the mRNA and protein levels in CC. (A) Scatter plot showing a strong positive correlation between YAP1 mRNA expression (RNA-seq RSEM) and YAP1 protein abundance (RPPA) in TCGA-CESC samples (Pearson r=0.85, p=1.24e-48). Each point represents a single tumor sample, colored by its YAP1 copy number alteration (CNA) status. (B) Box plot showing significantly elevated YAP1 protein expression in samples with YAP1 amplification compared to those with a diploid copy number (p=0.0007). Protein expression is presented as relative log2 expression from RPPA data. CESC, Cervical squamous cell carcinoma and endocervical adenocarcinoma; RPPA, Reverse phase protein array. [file Image1.jpeg]

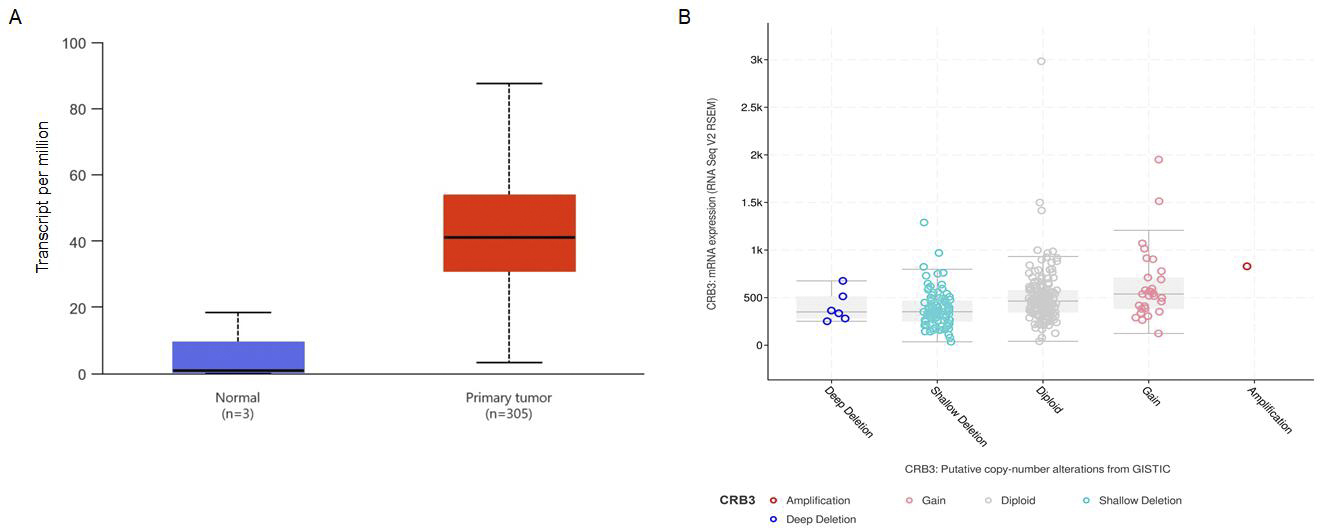

Supplement: Supplementary Figure 2 — CRB3 is overexpressed in CC and its expression shows a positive association with gene copy number alteration. (A) CRB3 mRNA expression is significantly up-regulated in primary CC tumors (n=305) compared to normal cervical tissues (n=3) in the TCGA-CESC cohort (p=0.0015). Expression values are shown as transcripts per million (TPM). (B) CRB3 mRNA expression shows a positive trend with increasing gene copy number. This trend reaches formal statistical significance when comparing tumors with CRB3 copy number gain to those with a diploid copy number (p=0.045). The elevated expression level in the single sample with CRB3 amplification (n=1) is consistent with this observed dose-response relationship. Expression values are derived from RNA-seq RSEM data. [file Image2.jpeg]

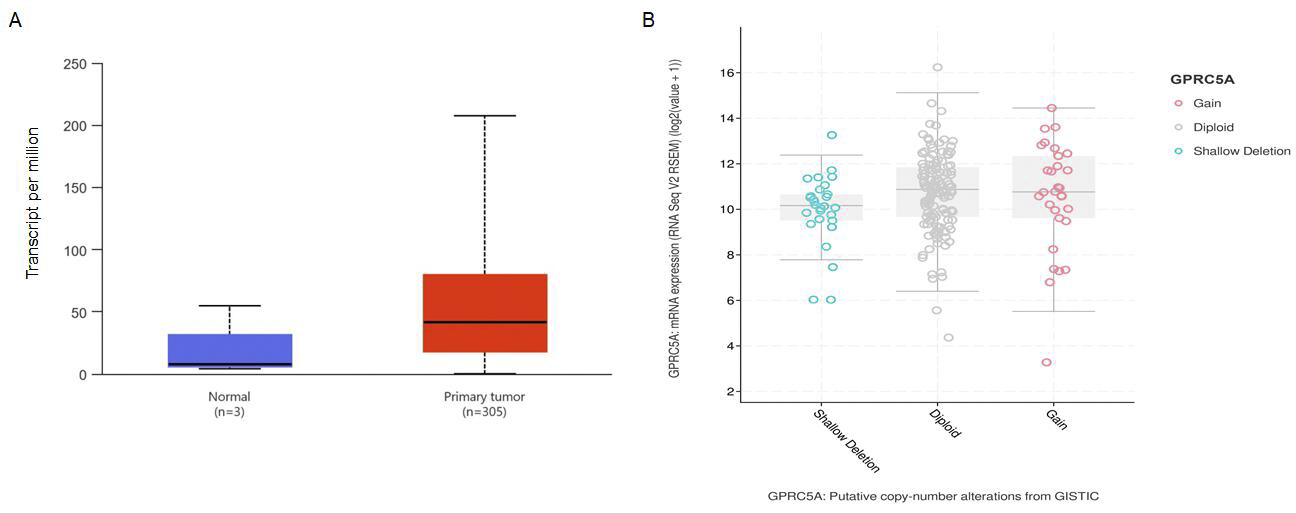

Supplement: Supplementary Figure 3 — GPRC5A expression in CESC and its correlation with gene copy number alteration. (A) GPRC5A mRNA expression in normal tissues (n=3) versus CESC primary tumors (n=305) from TCGA cohort. Data are presented as log2 (RSEM value+1). (B) GPRC5A mRNA expression levels across different copy number alteration groups: Gain, Diploid, and Shallow Deletion. The mRNA expression data (RNA Seq V2 RSEM, log2-transformed) are shown in relation to putative copy-number alterations determined by GISTIC. A Mann-Whitney U test revealed a statistically significant decrease in GPRC5A expression in the Shallow Deletion group compared to the Diploid group (p=0.025). [file Image3.jpeg]
