# Supplementary material for: Hippo signaling pathway in cervical cancer: insights into mechanisms and therapeutic potential
Source: Front Oncol. 2025 Nov 3;15:1662499. doi: 10.3389/fonc.2025.1662499 (PMC12620204; doi:10.3389/fonc.2025.1662499)
Supplement: Supplementary file 4 [file Table1.docx]

| **Supplementary Table 1 Summary of molecular alterations and functional roles of Hippo pathway components in CC** | | | | | | |
| --- | --- | --- | --- | --- | --- | --- |
| **Molecule Name** | **Relationship with Hippo Pathway** | Alteration in CC | | **Role in CC** | | **References** |
| YAP | Key transcriptional co-activator of the Hippo pathway, negatively regulated by the Hippo pathway | | Overexpression; Nuclear accumulation; Gene amplification (at 11q22) | | Enhances proliferation, migration, and invasion; synergizes with HR-HPV E6/E7 proteins to promote CC development | [17, 71-73, 86, 216] |
| TAZ | Homologous protein of YAP, functions overlap with YAP, negatively regulated by the Hippo pathway | | Overexpression; Gene amplification; Mutation (e.g., WWTR1 S89W) | | Promotes tumor growth, metastasis, and immune evasion; associated with PD-L1 expression | [106, 107, 155, 156] |
| LATS1/2 | Core kinases of the Hippo pathway, phosphorylate and inactivate YAP/TAZ | | Downregulation (LATS1 is downregulated in 45% of cases) | | Inhibits CC cell proliferation and invasion; LATS1 is often downregulated in CC | [87, 89, 94] |
| MST1/2 | Upstream kinases of the Hippo pathway, activate LATS1/2 | | Downregulation (STK4/MST1) | | Inhibits YAP/TAZ activity, exerts tumor-suppressive effects | [61, 198] |
| FAT1 | Upstream regulator, activates the Hippo pathway | | Inactivating mutation | | FAT1 mutation leads to YAP1 activation, promotes CC development | [38] |
| PTPN14 | Upstream regulator, inhibits YAP/TAZ activity | | Protein degradation (mediated by HPV E7 oncoprotein) | | Promotes YAP nuclear localization through HR-HPV E7 degradation and drives CC progression | [34, 98, 99] |
| AMOT | Regulates YAP activity by binding to LATS | | N/S | | Indirectly regulates the Hippo pathway, inhibits YAP activity | [28, 29] |
| CRB3 | Inhibits YAP activity by promoting LATS-mediated YAP phosphorylation | | N/S | | Inhibits YAP nuclear translocation and prevents tissue overgrowth | [30] |
| E-cadherin | Inhibits YAP by regulating MST1/2 activity and YAP/14-3-3 complex | | N/S | | Negatively regulates YAP, inhibits CC cell proliferation | [32, 33] |
| WWC2 | Inhibits YAP transcriptional activity, negatively regulates the Hippo pathway | | Downregulation (Targeted and suppressed by miR-146a-5p) | | miR-146a-5p activates YAP by targeting WWC2 and promotes CC metastasis | [200] |
| NEK2 | Inhibits the Hippo pathway by dephosphorylating MST1/2 and YAP | | Overexpression | | Promotes YAP nuclear accumulation, enhances CC cell proliferation and metastasis | [135, 143] |
| HIF-1α | Promotes cervical cancer cell proliferation and invasion by activating YAP/TAZ | | Overexpression (under hypoxic conditions) | | Activates YAP/TAZ under hypoxic conditions and promotes tumor progression | [147, 153] |
| PD-L1 | Regulated by YAP/TAZ and TEAD, involved in immune evasion | | Overexpression (driven by YAP/TAZ activity) | | High expression associated with CC progression and immune evasion | [107, 155, 156] |
| S100A7 | Regulated by YAP/TAZ and promotes tumor metastasis through EMT | | Overexpression | | Promotes CC cell migration and invasion | [161, 162] |
| AREG | Downstream target gene of YAP, activates EGFR | | Upregulation (transactivated by YAP) | | Forms a positive feedback loop and promotes CC cell proliferation and migration | [17] |
| TGF-α | Downstream target gene of YAP, activates EGFR | | Upregulation (transactivated by YAP) | | Synergizes with AREG to promote CC progression | [17] |
| NGF/TrkA | inhibits LATS1 and activates YAP | | Overexpression | | Promotes CC cell proliferation and migration | [133] |
| TBK1 | Inhibited by YAP/TAZ and affects antiviral immunity | | (Activity inhibited by YAP/TAZ; protein-level alteration not explicitly stated) | | YAP/TAZ enhances HPV infection by inhibiting TBK1 | [80] |

**Notes:**

**Molecule Name**: Molecules related to the Hippo pathway; **Relationship with Hippo Pathway**: The mechanism of the molecule in the Hippo pathway, such as activation, inhibition, or as a downstream target; **Alteration in CC:** The specific molecular-level changes observed in CC, such as overexpression, downregulation, gene amplification, inactivating mutation, or protein degradation; **Role in CC**: How the molecule affects the progression of CC, including promoting or inhibiting tumor growth, metastasis, immune evasion, etc; **References**: Specific reference numbers from the cited papers.
