# Supplementary material for: Hippo signaling pathway in cervical cancer: insights into mechanisms and therapeutic potential
Source: Front Oncol. 2025 Nov 3;15:1662499. doi: 10.3389/fonc.2025.1662499 (PMC12620204; doi:10.3389/fonc.2025.1662499)
Supplement: Supplementary file 5 [file Table2.docx]

| **Supplementary Table 2 The clinical potential of targeting Hippo pathway-interacting molecules in CC** | | | | | | |
| --- | --- | --- | --- | --- | --- | --- |
| Key  target (s) | Representative  drugs | Study  phase | Cancer  type | Treatment  regimen | Efficacy  results | Conclusion |
| EGFR | Cetuximab (168) | Phase II | CC | Cetuximab monotherapy: 400 mg/m² loading dose→250 mg/m² weekly maintenance | ORR：0%（90% CI 0–6.4%）; 6-month PFS rate: 14.3% (5/35); median PFS: 1.97 month; median OS: 6.7 month. All PFS-6 achievers had squamous histology | Cetuximab monotherapy was well-tolerated but demonstrated limited overall efficacy, with potential activity in squamous cell carcinomas |
|  | Erlotinib (170) | Phase II | CC | Erlotinib 150 mg/day (start 1 week before RT, continue until end of brachytherapy) + weekly cisplatin 40 mg/m² × 5 + pelvic RT 45 Gy/25 fx + brachytherapy 600 cGy × 4 fx | CR 94.4% (34/36), PR 5.6% (2/36), ORR 100%; 3-year OS 80.6%, 3-year PFS 73.8% (median follow-up 59.3 months) | E+CRT is feasible and safe, yielding unprecedented CR and long-term survival vs historical controls; EGFR-targeted chemoradiation is highly active in locally advanced CC and warrants further validation |
| PD-1 | Pembrolizumab (172) | Phase III | CC | Pembrolizumab 200 mg q3w + paclitaxel + cisplatin/carboplatin ± bevacizumab 15 mg/kg q3w (up to 35 cycles) | PD-L1 CPS≥1: With bevacizumab: median PFS 15.3 vs 10.3 mo (HR 0.56); median OS 43.9 vs 23.0 mo (HR 0.60) Without bevacizumab: median PFS 7.0 vs 6.0 mo (HR 0.61); median OS 17.5 vs 11.9 mo (HR 0.61) | Pembrolizumab-chemotherapy prolongs PFS & OS regardless of bevacizumab use; quadruplet is preferred when feasible, while pembrolizumab-chemotherapy alone remains standard-of-care for bevacizumab-ineligible patients |
|  | Cemiplimab (176) | Phase III | CC | Cemiplimab 350 mg q3w vs investigator’s choice of single-agent chemotherapy (pemetrexed, topotecan, irinotecan, gemcitabine, vinorelbine) | Median OS: 11.7 mo (Cemiplimab) vs 8.5 mo (Chemo), HR=0.67, p<0.00001; benefit seen regardless of PD-L1 status | Cemiplimab provides sustained OS benefit over chemotherapy regardless of PD-L1 status and has a manageable safety profile; should be considered standard second-line therapy |
| PD-L1 | Socazolimab (178) | Phase I | CC | Socazolimab 5 mg/kg intravenous every 2 weeks until progression | ORR 15.4 % (95% CI 8.7–24.5); median PFS 4.44 months (95% CI 2.37–5.75); median OS 14.72 months (95% CI 9.59–NE); ORR similar in PD-L1+ (16.7 %) and PD-L1– (17.9 %) patients; no treatment-related deaths | Socazolimab shows durable efficacy and a safety profile comparable to other PD-1/L1 mAbs in recurrent or metastatic CC |
| PI3Kα (encoded by PIK3CA) | Alpelisib (179) | Phase Ia  (First-in-Human) | Advanced solid tumors with PIK3CA mutations; CC (n=5 out of 134 total patients) | 400 mg once daily (established as the Maximum Tolerated Dose) | Overall Cohort: ORR 6.0% (8/134)  CC Cohort: ORR 60.0% (3/5 patients achieved a partial response) | Alpelisib demonstrated a manageable safety profile and promising preliminary efficacy in a biomarker-selected (PIK3CA-mutant) CC subgroup, providing a strong rationale for its further investigation in this population |

Note: ORR: Objective response rate; PFS: Progression-Free Survival; CI: Confidence interval; ORR: Objective response rate; OS: Overall survival; HR: Hazard ratio; GHS: Global health status; QoL: Quality of life; PF: physical functioning; NE: Not estimable; RT: Radiotherapy; Gy: Gray; fx: Fraction; cGy: Centigray; CR: Complete response; E+CRT: Erlotinib combined with cisplatin based chemoradiation; q3w: Every 3 weeks.
