# Supplementary material for: Hippo signaling pathway in cervical cancer: insights into mechanisms and therapeutic potential
Source: Front Oncol. 2025 Nov 3;15:1662499. doi: 10.3389/fonc.2025.1662499 (PMC12620204; doi:10.3389/fonc.2025.1662499)
Supplement: Supplementary file 6 [file Table3.docx]

| **Supplementary Table 3 Comparison of YAP and TAZ expression, function, and mechanisms in HPV16/18-positive CC** | | |
| --- | --- | --- |
| Category | YAP | TAZ |
| **Expression in HPV16+ CC** | Upregulated (17, 217) | Not significantly upregulated (217) |
| **Expression in HPV18+ CC** | Upregulated (17, 217) | Specifically and significantly upregulated (217) |
| **Key functional roles** | • Drives cell proliferation, invasion, and metastasis (17, 72) • Induces senescence in normal cervical epithelial cells (a tumor-suppressive function that is disrupted by HPV) (73) • Promotes immune evasion by inhibiting TBK1-mediated antiviral response (80) | • Independently drives carcinogenesis in HPV18+ CC (217) • Strongly promotes EMT, inhibits apoptosis, and expands cancer stem cell population (108) • Key driver of PD-L1 upregulation and immune evasion (107) |
| **Interplay with HPV oncoproteins** | • HPV E6: Prevents proteasomal degradation of YAP, leading to its stabilization and activation (17, 74) • HPV E7: Induces degradation of PTPN14, a negative regulator of YAP, promoting YAP nuclear localization (98, 99) | • HPV18 Context: TAZ operates independently of YAP; its specific upregulation is a hallmark of HPV18+ tumors (217) • Oncogenic Driver: TAZ activation is a critical driver in HPV18-associated carcinogenesis, with a distinct transcriptome from YAP (217) |
| Associated signaling pathways and key targets | • YAP-EGFR-AREG Feedback Loop: Forms a positive feedback circuit to drive malignancy (17) • YAP1-LATS2 Feedback Loop: A homeostatic mechanism disrupted by HPV E6/E7 (73) • YAP-TBK1 Axis: Suppresses innate antiviral immunity (80) | • TAZ-PD-L1 Axis: Directly upregulates PD-L1 expression to facilitate immune escape (107) • TAZ-TEAD: Activates a distinct transcriptional program critical for HPV18+ tumor growth (217) • Independent Pathways: Regulates a unique set of genes (e.g., TOGARAM2) not compensated by YAP (217) |
